# CircPPAP2B controls metastasis of clear cell renal cell carcinoma via HNRNPC-dependent alternative splicing and targeting the miR-182-5p/CYP1B1 axis

**DOI:** 10.1186/s12943-023-01912-w

**Published:** 2024-01-06

**Authors:** Zaosong Zheng, Xiangbo Zeng, Yuanchao Zhu, Mengxin Leng, Zhiyong Zhang, Qiong Wang, Xiaocen Liu, Siying Zeng, Yongyuan Xiao, Chenxi Hu, Shiyu Pang, Tong Wang, Bihong Xu, Peidan Peng, Fei Li, Wanlong Tan

**Affiliations:** 1 Department of Urology, Nanfang Hospital, Southern Medical University, Guangzhou, 510515 Guangdong China; 2 School of Traditional Chinese Medicine, Southern Medical University, Guangzhou, 510515 Guangdong China; 3 Department of Pathology, Nanfang Hospital, Southern Medical University, Guangzhou, 510515 Guangdong China

**Keywords:** CircPPAP2B, HNRNPC, m6A, Nuclear translocation, Alternative splicing, miRNA sponge

## Abstract

**Background:**

Renal cell carcinoma (RCC) is one of the most common malignant tumor worldwide. Metastasis is a leading case of cancer-related deaths of RCC. Circular RNAs (circRNAs), a class of noncoding RNAs, have emerged as important regulators in cancer metastasis. However, the functional effects and regulatory mechanisms of circRNAs on RCC metastasis remain largely unknown.

**Methods:**

High-throughput RNA sequencing techniques were performed to analyze the expression profiles of circRNAs and mRNAs in highly and poorly invasive clear cell renal cell carcinoma (ccRCC) cell lines. Functional experiments were performed to unveil the regulatory role of circPPAP2B in the proliferation and metastatic capabilities of ccRCC cells. RNA pulldown, Mass spectrometry analysis, RNA methylation immunoprecipitation (MeRIP), RNA immunoprecipitation (RIP), co-immunoprecipitation (CoIP), next-generation RNA-sequencing and double luciferase experiments were employed to clarify the molecular mechanisms by which circPPAP2B promotes ccRCC metastasis.

**Results:**

In this study, we describe a newly identified circular RNA called circPPAP2B, which is overexpressed in highly invasive ccRCC cells, as determined through advanced high-throughput RNA sequencing techniques. Furthermore, we observed elevated circPPAP2B in ccRCC tissues, particularly in metastatic ccRCC tissues, and found it to be associated with poor prognosis. Functional experiments unveiled that circPPAP2B actively stimulates the proliferation and metastatic capabilities of ccRCC cells. Mechanistically, circPPAP2B interacts with HNRNPC in a m6A-dependent manner to facilitate HNRNPC nuclear translocation. Subcellular relocalization was dependent upon nondegradable ubiquitination of HNRNPC and stabilization of an HNRNPC/Vimentin/Importin α7 ternary complex. Moreover, we found that circPPAP2B modulates the interaction between HNRNPC and splicing factors, PTBP1 and HNPNPK, and regulates pre-mRNA alternative splicing. Finally, our studies demonstrate that circPPAP2B functions as a miRNA sponge to directly bind to miR-182-5p and increase CYP1B1 expression in ccRCC.

**Conclusions:**

Collectively, our study provides comprehensive evidence that circPPAP2B promotes proliferation and metastasis of ccRCC via HNRNPC-dependent alternative splicing and miR-182-5p/CYP1B1 axis and highlights circPPAP2B as a potential therapeutic target for ccRCC intervention.

**Supplementary Information:**

The online version contains supplementary material available at 10.1186/s12943-023-01912-w.

## Background

Renal cell carcinoma (RCC) is a prevalent malignancy that has seen a notable increase in incidence over the last two decades [1, 2]. Clear cell renal cell carcinoma (ccRCC) is the most common histological subtype and represents approximately 75% of all RCC cases [3]. Even with improvements in early detection methods, approximately 30% of individuals diagnosed with RCC already exhibit distant metastases, while another 30% of those with localized or locally advanced RCC progress to metastatic RCC following surgical intervention [4]. Unfortunately, the 5-year survival rate of metastatic RCC patients is less than 10%, even with targeted therapy and immunotherapy [5, 6]. Hence, gaining a deeper insight into the molecular mechanisms driving RCC metastasis is of utmost importance in the search to discover new therapeutic targets and enhance the overall prognosis for patients [7, 8].

Circular RNAs (circRNAs) represent a class of noncoding RNAs with single-stranded and closed-loop structures produced by precursor mRNA (pre-mRNA) back splicing of exons [9–11]. A body of evidence revealed that circRNAs play critical biological functions in cancer progression, including gene transcription regulation, miRNAs sponges, RNA-binding protein interaction, and protein translation templates [12–15]. For example, CircPVT1 can act as competing endogenous RNA and promote ccRCC progression by directly binding to miR-145-5p and regulating TBX15 expression [16]. CircNDUFB2 inhibits non-small cell lung cancer progression through interacting and destabilizing IGF2BPs [17]. C-E-Cad, encoded by a circular RNA circ-E-cad, is overexpressed in Glioblastoma and activates EGFR through interaction with the EGFR CR2 domain, thereby maintaining glioma stem cell tumorigenicity [18]. However, the functional effects and regulatory mechanisms of circRNAs on ccRCC metastasis remain largely unknown [19, 20].

Interaction with RNA-binding protein is an important regulatory mechanism of circRNAs. Heterogeneous nuclear ribonucleoprotein C (HNRNPC) has been reported as an m6A reader and functions as an RNA-binding protein to recognize m6A modified RNA in cancer metastasis. HNRNPC contributes to multiple aspects of the physiological and pathological process, including alternative splicing, transcriptional regulation, and translational regulation [21, 22]. However, it is still unclear how HNRNPC is regulated. Further insight into the role and underlying mechanism of HNRNPC could provide valuable knowledge regarding ccRCC metastasis and contribute to the development of targeted therapies [23].

In this study, we identify a novel circRNA, circPPAP2B, which is significantly upregulated in highly invasive ccRCC cells and metastatic ccRCC tissues. Functional investigations revealed that circPPAP2B promotes ccRCC proliferation and metastasis in vitro and in vivo. Mechanistically, circPPAP2B directly binds to HNRNPC in a m6A-dependent manner, triggering the nuclear translocation of HNRNPC via its interaction with multiple proteins involved in ubiquitination and nuclear import. We found that circPPAP2B regulates the interaction between HNRNPC and splicing factors and affects pre-mRNA alternative splicing. Furthermore, we report that circPPAP2B also functions as a miRNA sponge, and directly binds to miR-182-5p to regulate CYP1B1 expression. Therefore, our data demonstrate that the newly identified circPPAP2B promotes the proliferation and metastasis of ccRCC via HNRNPC-dependent alternative splicing and targeting the miR-182-5p/CYP1B1 axis.

### Methods

All methods and materials are described in the Supplementary Methods.

## Results

### Identification of circPPAP2B in highly invasive ccRCC cell lines

**Fig. 1:**
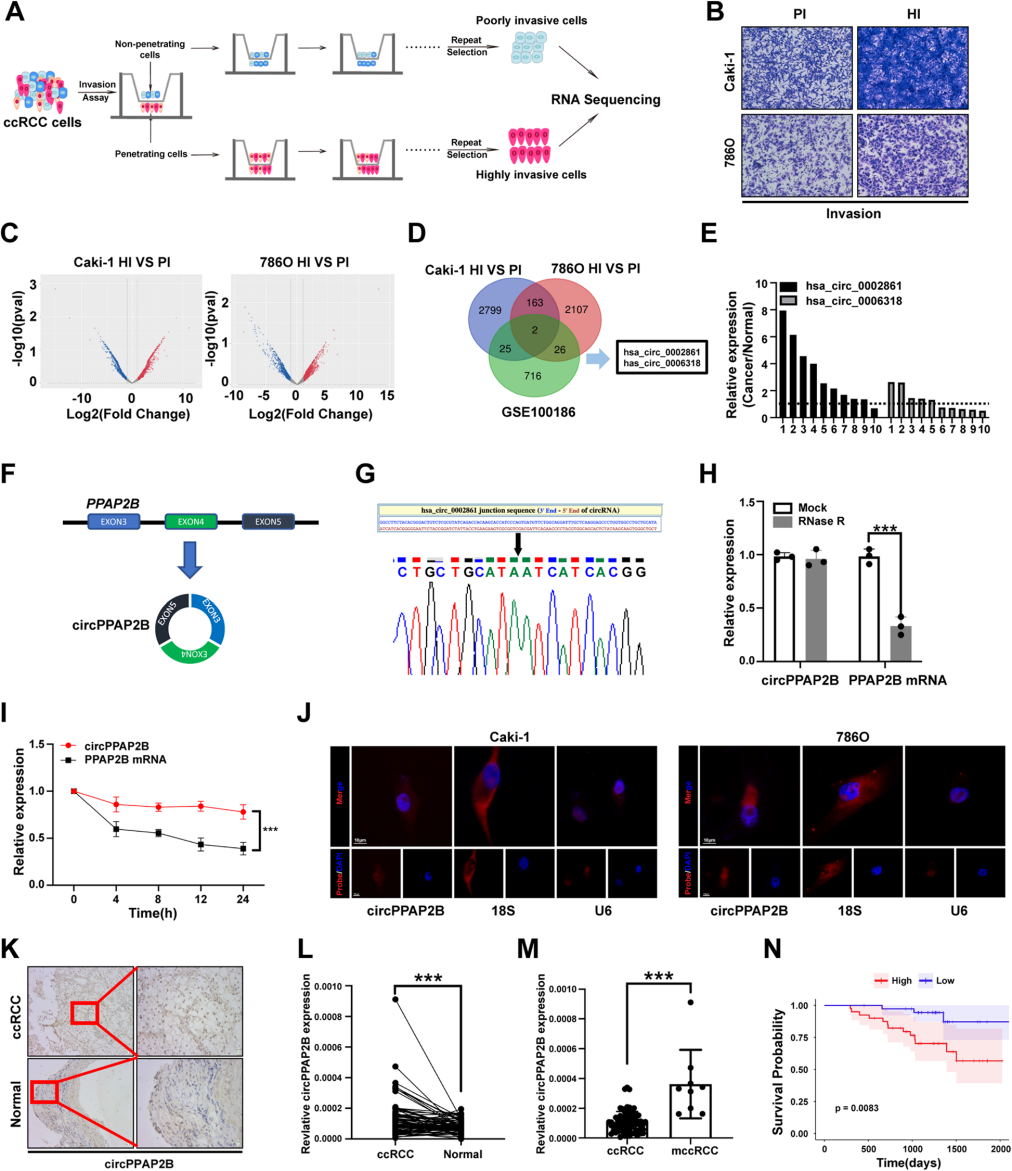
Identification of circPPAP2B in highly invasive ccRCC cell lines. **A** Establishment of highly invasive and poorly invasive ccRCC cell lines. The highly invasive and poorly invasive ccRCC cell lines were generated through experimental protocols described in the Methods section. **B** Assessment of the invasive capabilities of invasive and poorly invasive ccRCC cell lines using transwell assay. Representative images of invasive cells and quantification of invasive cell numbers are shown. **C** High-throughput RNA sequencing to analyze the expression profiles of circRNAs in highly and poorly invasive ccRCC cell lines. **D** A Venn diagram illustrates the overlapping upregulated circRNAs between highly and poorly invasive ccRCC cell lines. **E** qPCR validated the upregulation of circPPAP2B in ccRCC tissues. **F** A schematic illustration of circPPAP2B originates from exons 3 to 5 of the *PPAP2B* gene. **G** Sanger sequencing validated the circPPAP2B junction site in circBase. **H** RNase R assay evaluates the stability of circPPAP2B in the ccRCC cell line.** I** Actinomycin D assay evaluates the stability of circPPAP2B in the ccRCC cell line. **J** RNA FISH was performed to determine the subcellular localization of circPPAP2B in ccRCC cell lines. **K** RNA FISH was performed to detect circPPAP2B expression in ccRCC tissues and normal tissues. **L-M** qPCR was performed to detect circPPAP2B expression on a cohort of 78 pairs of ccRCC tissues and adjacent non-tumorous tissues. **N** Kaplan–Meier survival analysis was conducted to evaluate the prognostic value of circPPAP2B in ccRCC. Data are represented as mean ± SEM. ***P < 0.001 vs. WT group

Multiple studies have shown an important role for circRNAs in the process of cancer metastasis, including in ccRCC [11, 24]. To investigate the role of circRNAs in the invasive potential of ccRCC, we isolated two distinct ccRCC cell lines, 7860-HI (highly invasive) and 7860-PI (poorly invasive), as well as Caki-1-HI (highly invasive) and Caki-1-PI (poorly invasive) by repeated transwell assays (Fig. 1A). To assess the invasive capabilities of these cell lines, we conducted a transwell assay. Results demonstrated that both 7860-HI and Caki-1-HI cell lines exhibited higher invasive ability compared to 7860-PI and Caki-1-PI cell lines (Fig. 1B). We next proceeded to perform high-throughput RNA sequencing to analyze the expression profiles of circRNAs and mRNAs in highly and poorly invasive ccRCC cell lines (Fig. 1C, Figure S1A-B). Gene ontology (GO) enrichment analysis revealed that differentially expressed genes between highly invasive and poorly invasive ccRCC cells are enriched in several biological processes, including cell adhesion, regulation of cell growth, extracellular matrix, and plasma membrane (Figure S1C-D). KEGG pathway analysis revealed these differentially expressed genes are enriched in significant pathways, including pathways in cancers, microRNAs in cancer, and cell adhesion molecules (Figure S1E-F). By overlapping our results with the publicly available GSE100186 dataset, we identified a total of two upregulated circRNAs [25] (Fig. 1D and Supplementary Table 1). To validate the expression of these candidate circRNAs, we performed the quantitative reverse transcription PCR (qPCR) in 10 paired ccRCC tissues. The results revealed that hsa_circ_0002861, derived from the *PPAP2B* gene, thereafter named circPPAP2B, was significantly upregulated in ccRCC tissues when compared to adjacent non-tumoral tissues (Fig. 1E). Taken together, our data strongly suggested that circPPAP2B is upregulated in ccRCC and may be associated with the highly invasive nature of ccRCC.

### Characteristics of circPPAP2B in ccRCC cells

We further characterized circPPAP2B and explored its subcellular localization in ccRCC. The mature sequence of circPPAP2B spans 512 bp and originates from exons 3 to 5 of the *PPAP2B* gene (Fig. 1F). To confirm the presence of the backsplicing junction site in circPPAP2B, Sanger sequencing was performed using divergent primers and the result showed the circPPAP2B junction sequences were completed by circBase [26] (Fig. 1G). To evaluate the stability of circPPAP2B, we employed RNase R, an exonuclease known for its ability to selectively degrade linear mRNA. As expected, the linear mRNA level of PPAP2B was significantly reduced upon treatment with RNase R, whereas circPPAP2B expression remained unaffected (Fig. 1H). This data confirms that circPPAP2B is resistant to RNase R and enhanced stability compared to linear PPAP2B mRNA. To assess the half-life of circPPAP2B, ccRCC cells were treated with transcriptional inhibitor actinomycin D for 24 h. These studies revealed that circPPAP2B exhibited an extended half-life when compared to linear PPAP2B mRNA (Fig. 1I). We next determine the subcellular localization of circPPAP2B using RNA fluorescence in situ hybridization (FISH) assay. The results indicated that circPPAP2B is distributed in both the nucleus and cytoplasm of ccRCC cells (Fig. 1J). Collectively, our findings confirm the high stability and subcellular localization of circPPAP2B in ccRCC cells.

### CircPPAP2B is overexpressed in ccRCC and correlates with metastasis

To explore the biological function of circPPAP2B in ccRCC, we investigate its expression in various ccRCC cell lines. The results demonstrated that ccRCC cell lines exhibited higher expression of circPPAP2B compared to immortalized human renal proximal tubule epithelial cells Human Kidney-2 (HK-2), indicating a potential association between circPPAP2B and ccRCC (Figure S2A). Importantly, RNA FISH assays and qPCR revealed that highly invasive cell lines displayed elevated expression of circPPAP2B (Figure S2B-C). To gain insight into the expression pattern of circPPAP2B in ccRCC, we conducted RNA FISH in ccRCC tissue and normal tissue. The result revealed that circPPAP2B expression is higher in ccRCC tissues than in normal tissue (Fig. 1K). We next analyzed circPPAP2B expression using qPCR on a cohort of 78 pairs of ccRCC tissues and adjacent non-tumorous tissues. The results demonstrated a significant increase in circPPAP2B expression in ccRCC tissues when compared to adjacent non-tumorous tissues (Fig. 1L). Importantly, within the ccRCC tissues, those with distant metastasis exhibited higher circPPAP2B expression than those without distant metastasis (Fig. 1M). To evaluate the diagnostic potential of circPPAP2B in its ability to discriminate ccRCC associated with or without distant metastasis, receiver operating characteristic (ROC) analysis was performed. Notably, circPPAP2B expression showed a favorable area under the curve (AUC) of 0.94, indicating its potential as a reliable marker for differentiating between ccRCC with and without distant metastasis (Figure S2D). Finally, ccRCC patients were divided into high-circPPAP2B-expression group and low-circPPAP2B-expression group according to the median expression. Kaplan–Meier survival analysis indicated a significant difference between the high-circPPAP2B-expression group and the low-circPPAP2B-expression group, confirming a prognostic value of circPPAP2B in ccRCC (Fig. 1N).

### CircPPAP2B promotes ccRCC proliferation and metastasis

**Fig. 2:**
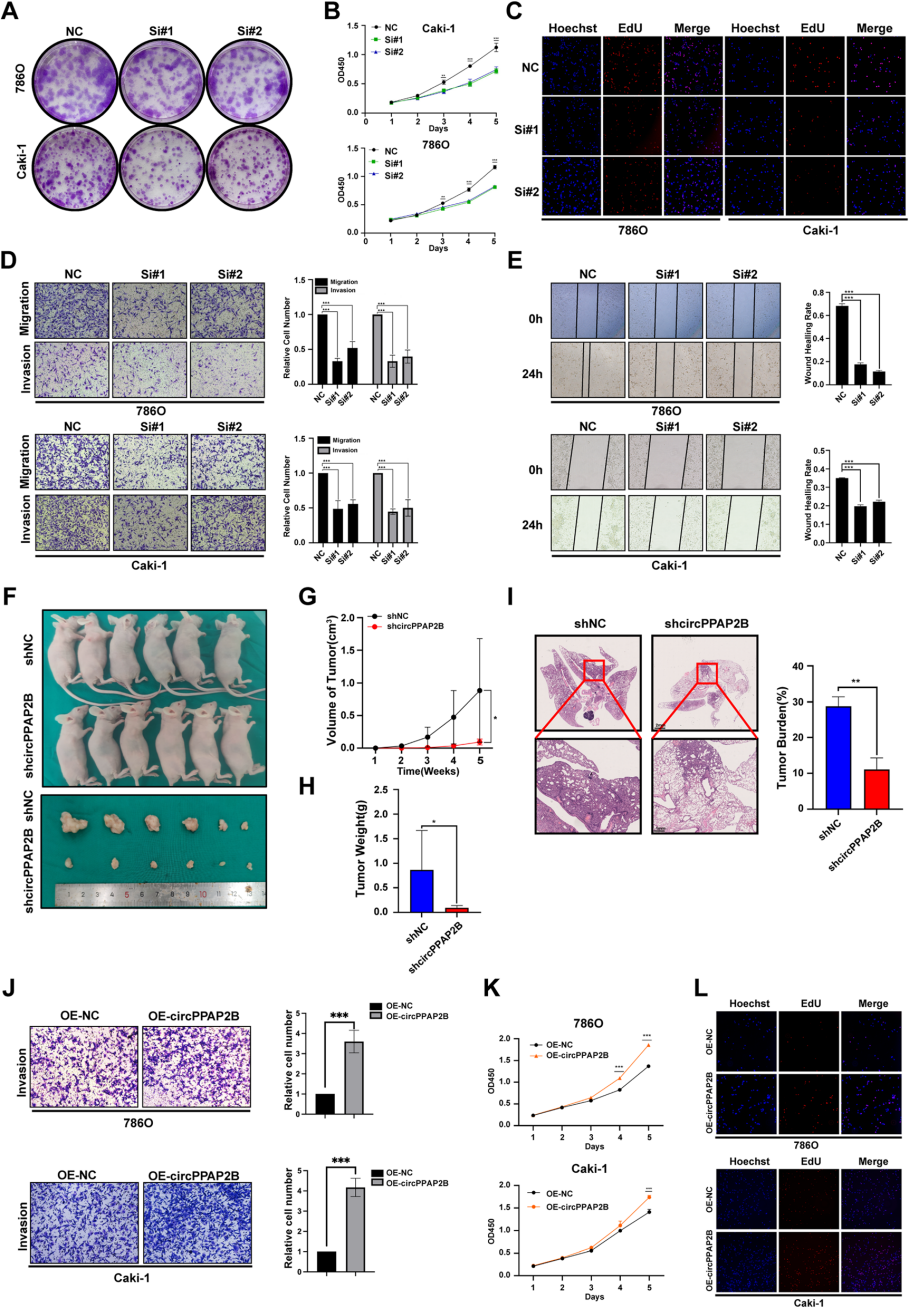
CircPPAP2B promotes ccRCC proliferation and metastasis in vivo and in vitro **A-C** Colony formation assays, CCK8 assays, and Edu assays were performed to evaluate the proliferation ability of ccRCC cells treated with shNC or shcircPPAP2B. **D-E** Transwell assays and wound-healing assays were conducted to detect the invasive ability of ccRCC cells treated with shNC or shcircPPAP2B. **F **The representative images of nude mice subcutaneously injected with caki-1 stably expressed shNC or shcircPPAP2B. **G** The volume of tumors was measured every week. **H **The weight of tumors was measured 5 weeks after injection. **I** The representative images of lung metastases in nude mice by HE staining. Tumor burdens were reflected by the percentages of tumor area in each slide. **J** Transwell assays were conducted to detect the invasive ability of ccRCC cells treated with OE-NC or OE-circPPAP2B. **K-L** CCK8 assays and Edu assays were performed to evaluate the proliferation ability of ccRCC cells treated with OE-NC or OE-circPPAP2B. Data are represented as mean ± SEM. *P < 0.05, **P < 0.01, ***P < 0.001 vs. WT group

To investigate the biological significance of circPPAP2B in ccRCC, we designed and synthesized siRNAs specifically targeting the circPPAP2B junction site. The efficiency of circPPAP2B knockdown was confirmed by qPCR (Figure S3A). Colony formation assays, CCK8 assays, and Edu assays were conducted to evaluate the effect of circPPAP2B knockdown on the proliferation of ccRCC cells. Remarkably, we observed a significant reduction in cell proliferation upon circPPAP2B silencing (Fig. 2A-C). Moreover, transwell migration assays, invasion assays, and wound-healing assays all demonstrated that circPPAP2B knockdown significantly inhibited the migration and invasiveness of ccRCC cells (Fig. 2D-E). To further validate our in vitro findings, we next performed subcutaneous and blood xenograft assays in vivo. Caki-1 cells stably expressing shcircPPAP2B or control vector were subcutaneously injected into nude mice. Tumor volume was measured weekly, and tumor weight was assessed five weeks after injection. Strikingly, we observed substantially smaller tumor volumes and lighter tumor weights in the shcircPPAP2B group when compared to the vector control group. These results are consistent with our in vitro observations and further demonstrate that circPPAP2B knockdown significantly inhibited subcutaneous xenograft tumor growth (Fig. 2F-H). We next investigated the metastasis potential of circPPAP2B in vivo. To this end we injected Caki-1 cells stably expressing shcircPPAP2B or a control vector into nude mice via their tail veins. Eight weeks after injection, the mice were sacrificed, and HE staining was performed. Our studies revealed a significant reduction in the lung metastatic burden upon circPPAP2B knockdown (Fig. 2I). To further confirm these results, we constructed a circPPAP2B overexpression vector to test the effect of increased expression of circPPAP2B in ccRCC cells. Overexpression circPPAP2B was confirmed by qPCR (Figure S3B-C). Consistent with results from shRNA studies, circPPAP2B overexpression significantly increased the invasion capacity of ccRCC cells in transwell invasion assays (Fig. 2J). In addition, circPPAP2B overexpression also significantly increased the proliferation of ccRCC cells in CCK8 assays and Edu assays (Fig. 2K-L). These findings conclusively demonstrate that circPPAP2B promotes ccRCC proliferation and metastasis both in vitro and in vivo.

### CircPPAP2B directly interacts with HNRNPC in a m6A-dependent manner

**Fig. 3:**
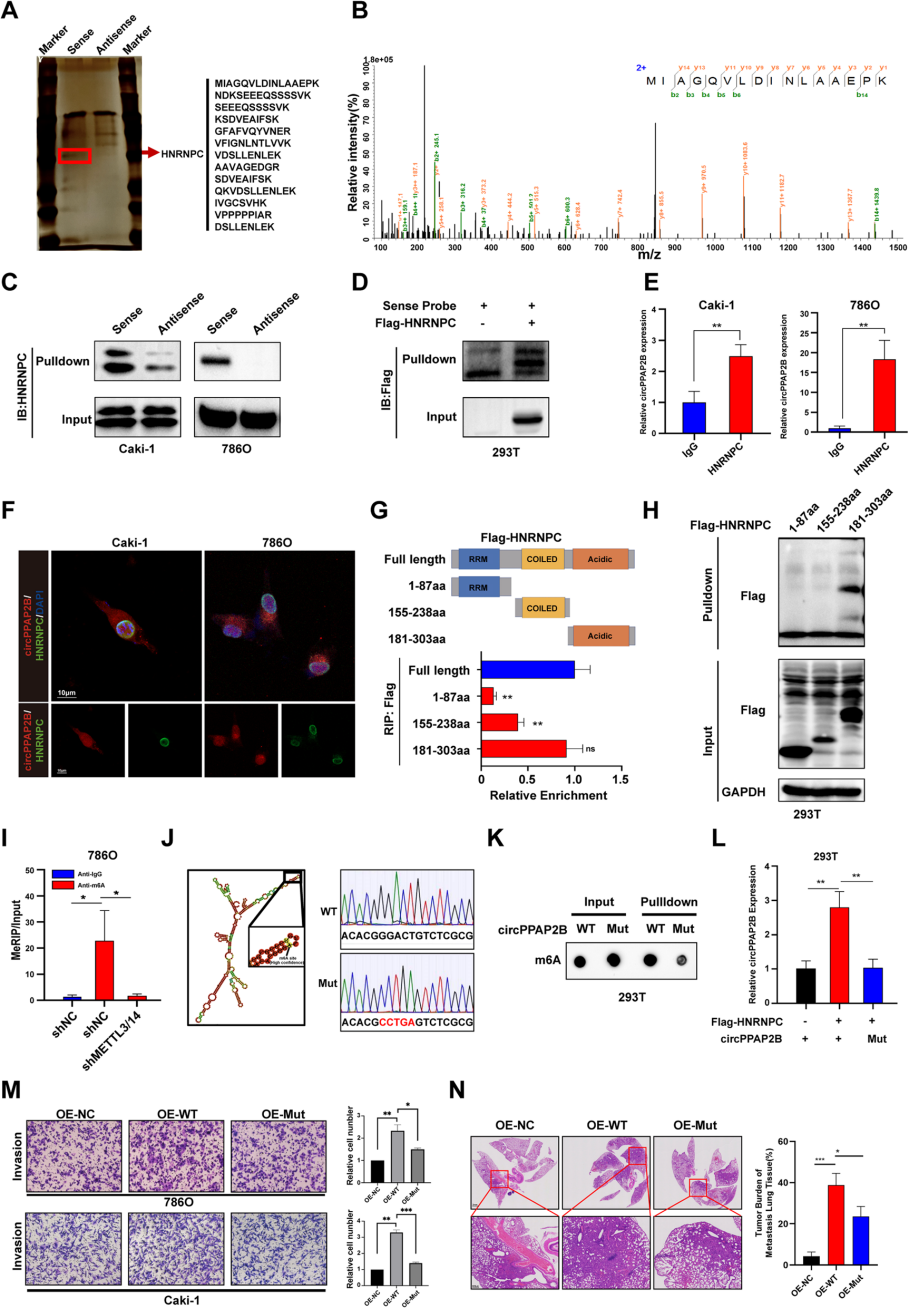
CircPPAP2B directly interacts with HNRNPC in a m6A-dependent manner **A**. RNA pulldown assay was performed using a biotin-labeled sense or antisense probe of circPPAP2B. The proteins enriched by sense or antisense probe of circPPAP2B were separated by SDS-PAGE and subjected to silver staining. **B** Mass spectrometry analysis identified HNRNPC as one of the most abundant proteins enriched by the sense probe of circPPAP2B. **C** RNA pulldown assay was performed to confirm the direct interaction between circPPAP2B and HNRNPC in ccRCC cell lines. **D** RNA pulldown assay was performed to confirm the direct interaction between circPPAP2B and HNRNPC in 293 T. **E** RIP assay was performed to validate direct interaction between circPPAP2B and HNRNPC in ccRCC cell lines using anti-HNRNPC antibody. **F** RNA FISH was performed to determine the co-localization of circPPAP2B and HNRNPC in ccRCC cell lines. **G** Full-length and truncated flag-labeled HNRNPC was constructed and RIP assays were performed to identify the specific domain of HNRNPC which interacts with circPPAP2B. **H** RNA pulldown assays were performed to identify the specific domain of HNRNPC which interacts with circPPAP2B. **I** MeRIP assays were performed to investigate whether circPPAP2B undergoes m6A modification. **J** Graphic illustration of the secondary structure of circPPAP2B and its m6A modification site predicted by RNA-fold software. Construction of WT and m6A modification site mutant circPPAP2B. **K** RNA pulldown assay and dot blot assay were performed to validate the m6A modification site in circPPAP2B. **L** RIP assay was performed to validate the m6A modification site in circPPAP2B and its role in the interaction between circPPAP2B and HNRNPC. **M** Transwell assay was performed on the role of m6A modification site in circPPAP2B in promoting ccRCC invasion. **I** The representative images of lung metastases in nude mice by HE staining. Tumor burdens were reflected by the percentages of tumor area in each slide. Data are represented as mean ± SEM. *P < 0.05, **P < 0.01, ***P < 0.001 vs. WT group

Previous studies have suggested that circRNAs can function as miRNA sponges, RNA binding proteins (RBPs) interacting RNA, and serve as protein translation templates. To evaluate the potential protein translational of circPPAP2B, we utilized a bioinformatics tool to predict its open reading frames (ORFs) and internal ribosome entry site (IRES) [27]. These results revealed that there was no IRES sequence in circPPAP2B, indicating a lack of translational potential (Figure S3D and Supplementary Table 2). To explore whether circPPAP2B interacts with proteins to promote metastasis in ccRCC, we conducted an RNA pulldown assay using a biotin-labeled sense or antisense probe of circPPAP2B transfected into ccRCC cells Caki-1. The proteins enriched by the pulldown assay were separated by Sodium Dodecyl Sulfate PolyAcrylamide Gel Electrophoresis (SDS-PAGE) and subjected to silver staining (Fig. 3A). Mass spectrometry analysis of the specific band at approximately 40 kDa in the sense probe group identified HNRNPC as one of the most abundant proteins with a high score and abundance (Fig. 3B and Supplementary Table 3). The direct interaction between circPPAP2B and HNRNPC was confirmed by RNA pulldown assay followed by western blotting in Caki-1, 786O, and 293 T (Fig. 3C-D). Importantly, this interaction was also confirmed using an RNA immunoprecipitation (RIP) assay with an anti-HNRNPC antibody (Fig. 3E). Finally, RNA FISH was performed to confirm the co-location of circPPAP2B and HNRNPC (Fig. 3F). In order to map the region of HNRNPC that interacts with circPPAP2B, we constructed a full-length and truncated flag-labeled HNRNPC vector based on its structural domain sequences and performed RNA pulldown and RIP assays [28]. The results revealed that circPPAP2B directly interacts with the Acidic Region of HNRNPC, and not with the RRM or Coiled-coil Region (Fig. 3G-H).

Previous studies have proposed that HNRNPC might act as a N6-methyladenosine (m6A) reader and interact with m6A-modified RNA [29]. To investigate whether circPPAP2B undergoes m6A modification, we conducted m6A RNA immunoprecipitation (MeRIP) assays. Significantly, enriched m6A modification was detected in circPPAP2B. Knockdown of core components of the multicomponent methyltransferase complex (MTC), including METTL3 and METTL14, resulted in a significant reduction of m6A modification in circPPAP2B (Fig. 3I). Given that m6A modification preferentially occurred on the RRACH consensus sequence (R = A/G, H = A/C/U), we predicted a potential m6A modification site (GGACU) in the circPPAP2B sequence [30]. We constructed wild-type (WT) and mutant (Mut) circPPAP2B and validated it by Sanger sequencing (Fig. 3J). The mutations in the m6A modification site remarkably reduced the m6A modification in circPPAP2B and significantly impaired the interaction between circPPAP2B and HNRNPC (Fig. 3K-L). Importantly, WT circPPAP2B promoted invasion of ccRCC cells, whereas Mut circPPAP2B failed to exhibit a pro-invasion effect (Fig. 3M). Moreover, WT circPPAP2B, rather than Mut circPPAP2B, dramatically increased lung metastatic burden in the in vivo metastasis assays (Fig. 3N). Collectively, these data revealed that CircPPAP2B directly interacts with HNRNPC in a m6A-dependent manner to promote the invasiveness of ccRCC cells.

### CircPPAP2B stabilizes HNRNPC/Vimentin/Importin α7 interaction to facilitate HNRNPC nuclear translocation

**Fig. 4:**
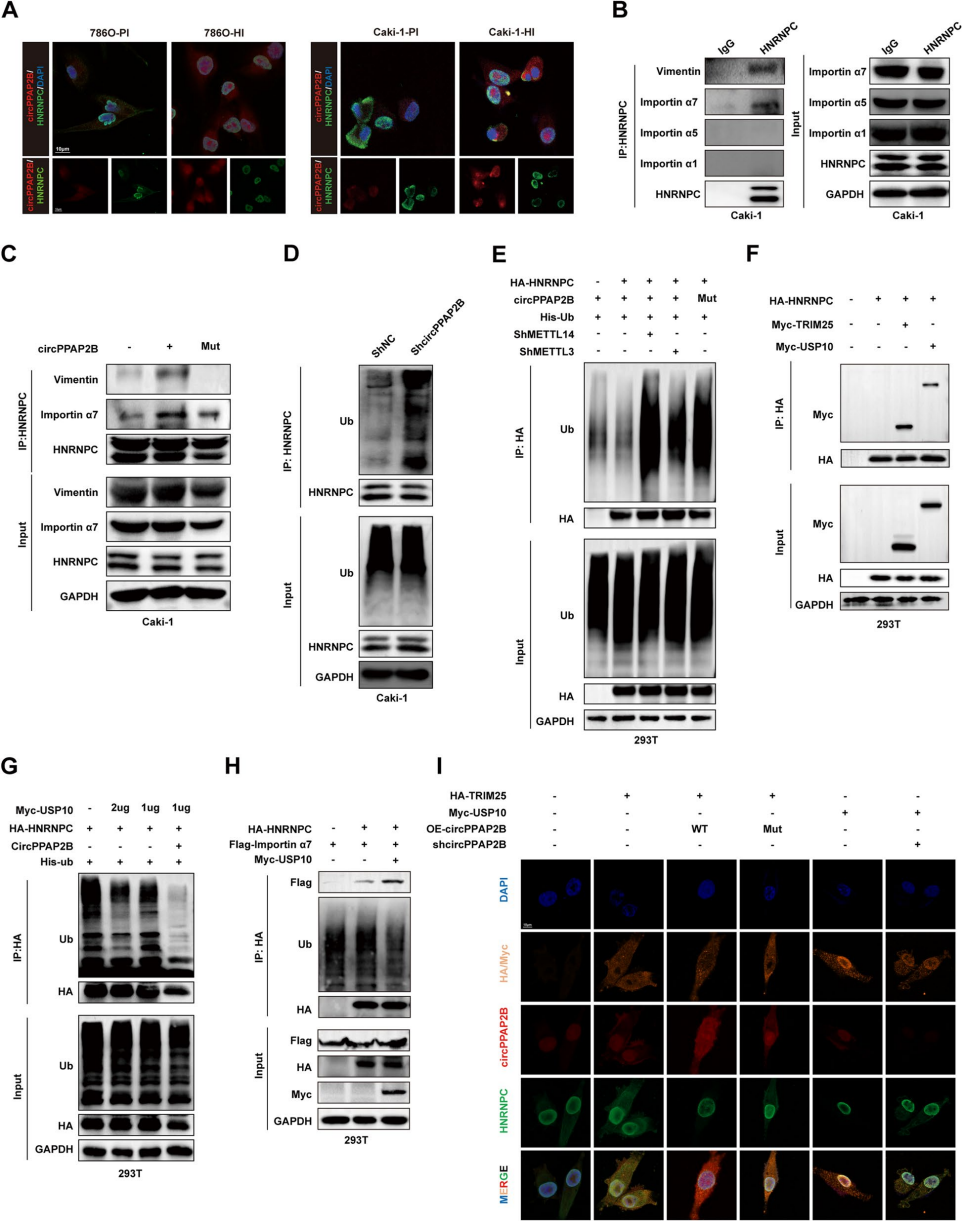
CircPPAP2B facilitates HNRNPC nuclear translocation via regulating HNRNPC nondegradable ubiquitination and stabilizing HNRNPC/Vimentin/Importin α7 ternary complex **A** RNA FISH was performed to determine subcellular localization of HNRNPC in highly and poorly invasive ccRCC cell lines. **B** CoIP assay was performed to determine the direct binding between HNRNPC with Vimentin and Importin α. **C** Endogenous CoIP assay was performed to confirm the direct interaction between HNRNPC and Vimentin with Importin α7, and the role of the m6A modification site in their interaction. **D** CoIP assay was performed to explore whether circPPAP2B regulates the ubiquitination of HNRNPC in ccRCC cell lines. **E** CoIP assay was performed to determine whether the regulatory of circPPAP2B on HNRNPC ubiquitination is dependent on m6A modification. **F** CoIP assay was performed to confirm the direct interaction between HNRNPC and TRIM25 or USP10. **G** CoIP assay was performed to determine the role of USP10 on ubiquitination levels of HNRNPC. **H** CoIP assay was performed to explore whether HNRNPC ubiquitination regulates the interaction between HNRNPC and Importin α7.** I** FISH was performed to determine the role of TRIM25 or USP10 on the subcellular localization of HNRNPC in ccRCC cells

Since circRNAs have been shown to influence protein subcellular localization [31], we investigated the localization of HNRNPC, which appeared mostly in the nucleus of 786O-HI and Caki1-HI but excluded from nuclei of 786O-PI and Caki1-PI cells (Fig. 4A). Consistent with these observations, the depletion of endogenous circPPAP2B using shRNA significantly reduced the nuclear localization of HNRNPC (Figure S4A). In further support of our findings, circPPAP2B overexpression reversed the shRNA circPPAP2B-induced downregulation on HNRNPC nuclear localization, while the mutated form of circPPAP2B did not (Figure S4A).

Previous studies have highlighted the significant role of cytoskeletons in protein plasmic transportation [32]. Our Mass Spectrometry analyses revealed the interaction between HNRNPC and Vimentin, an important cytoskeleton protein involved in epithelial-to-mesenchymal transition (EMT) and cancer metastasis (Figure S4B). The interaction between HNRNPC and Vimentin was further validated in CoIP assays (Fig. 4B). Since circPPAP2B affected the nuclear localization of HNRNPC, we next investigated the potential role of importin α proteins, which have been reported to play important roles in the nucleocytoplasmic transport of proteins. Using CoIP assay we found that HNRNPC specifically interacts with Importin α7, but not with Importin α1 or Importin α5 (Fig. 4B). Additional exogenous CoIP assay further confirmed the direct interaction between HNRNPC and Importin α7 (Figure S4C-E). Notably, depletion of endogenous circPPAP2B using shRNA significantly reduced the interaction between HNRNPC and Importin α7, while circPPAP2B overexpression increased this interaction (Figure S4F). Using full-length or truncated flag-labeled HNRNPC and HA-labeled Importin α7, we identified the binding site between HNRNPC and Importin α7 between amino acid 181–238 (Figure S4G). Based on our finding that the m6A modification site of circPPAP2B regulated the interaction between circPPAP2B and HNRNPC, we further examined whether the m6A modification site impacted the HNRNPC/Vimentin/Importin α7 interaction. CircPPAP2B overexpression significantly increase the HNRNPC/Vimentin/Importin α7 interaction. However, these effects were not observed when the mutated form of circPPAP2B was overexpressed (Fig. 4C). Taken together, these findings suggest that circPPAP2B plays a critical role in the nuclear translocation of HNRNPC by stabilizing the HNRNPC/Vimentin/Importin α7 complex interactions.

### Nondegradable ubiquitination is critical for HNRNPC nuclear translocation

We next explored the regulatory mechanism of circPPAP2B on HNRNPC nuclear translocation. We found no significant changes in the half-life of HNRNPC between circPPAP2B knockdown and control cells (Figure S5A). Consistently, both circPPAP2B knockdown and circPPAP2B overexpression did not lead to substantial alterations in HNRNPC protein expression levels (Figure S5B). Since circRNAs have been reported to interact with RNA-binding proteins and regulate their ubiquitination [17], we investigated whether circPPAP2B regulates the ubiquitination of HNRNPC. Interestingly, the depletion of circPPAP2B resulted in a significant increase in the ubiquitination of HNRNPC in both ccRCC cells and 293 T cells (Fig. 4D and S5C). Moreover, deletion of METTL3/METTL14 or mutation of the m6A modification site in circPPAP2B also significantly enhanced HNRNPC ubiquitination. Together, these results suggest that circPPAP2B regulates HNRNPC ubiquitination in an m6A modification-dependent manner (Fig. 4E). To identify the E3 ligases or deubiquitinases (DUBs) that may be involved in regulating HNRNPC ubiquitination. Mass Spectrometry data analyses identified TRIM25 and USP10 as potential regulators (PXD029560). TRIM25 is a member of the tripartite motif family of E3 ubiquitin ligases, responsible for adding polyubiquitin chains to substrates, while USP10 is a member of ubiquitin-specific protease family, involved in removing polyubiquitin chains from substrates. CoIP assay confirmed interactions between TRIM25 or USP10 and HNRNPC (Fig. 4F). Notably, circPPAP2B overexpression further increased interactions between USP10 and HNRNPC but significantly reduced interactions between TRIM25 and HNRNPC. However, these effects were not observed when the mutated form of circPPAP2B was overexpressed (Figure S5D-F).

Furthermore, ubiquitination levels of HNRNPC were significantly decreased following USP10 overexpression, while circPPAP2B overexpression dramatically promoted USP10-induced HNRNPC deubiquitination (Fig. 4G). In contrast, ubiquitination levels of HNRNPC were significantly increased with TRIM25 overexpression (Figure S5G). We found that USP10 dramatically reduced the ubiquitination levels of HNRNPC and increased the interaction between HNRNPC and Importin α7, while TRIM25 dramatically decreased the interaction between HNRNPC and Importin α7 (Fig. 4H and S5H). These reults suggest that HNRNPC ubiquitination is an important regulatory mechanism for its nuclear translocation. In fact, FISH assays further revealed that circPPAP2B overexpression reversed TRIM25-induced inhibition of HNRNPC nuclear translocation, while overexpression of mutant circPPAP2B failed to exhibit a similar effect. Consistently, USP10 overexpression increased nuclear translocation of HNRNPC, which was efficiently reversed by circPPAP2B knockdown (Fig. 4I). Altogether, these results demonstrate that TRIM25 and USP10 regulate HNRNPC ubiquitination and nuclear translocation. Our data further demonstrate that interaction between HNRNPC and TRIM25 or USP10 is regulated by circPPAP2B in a m6A-dependent manner.

### HNRNPC recruits PTBP1 and HNRNPK to regulate pre-mRNA alternative splicing in ccRCC

**Fig. 5:**
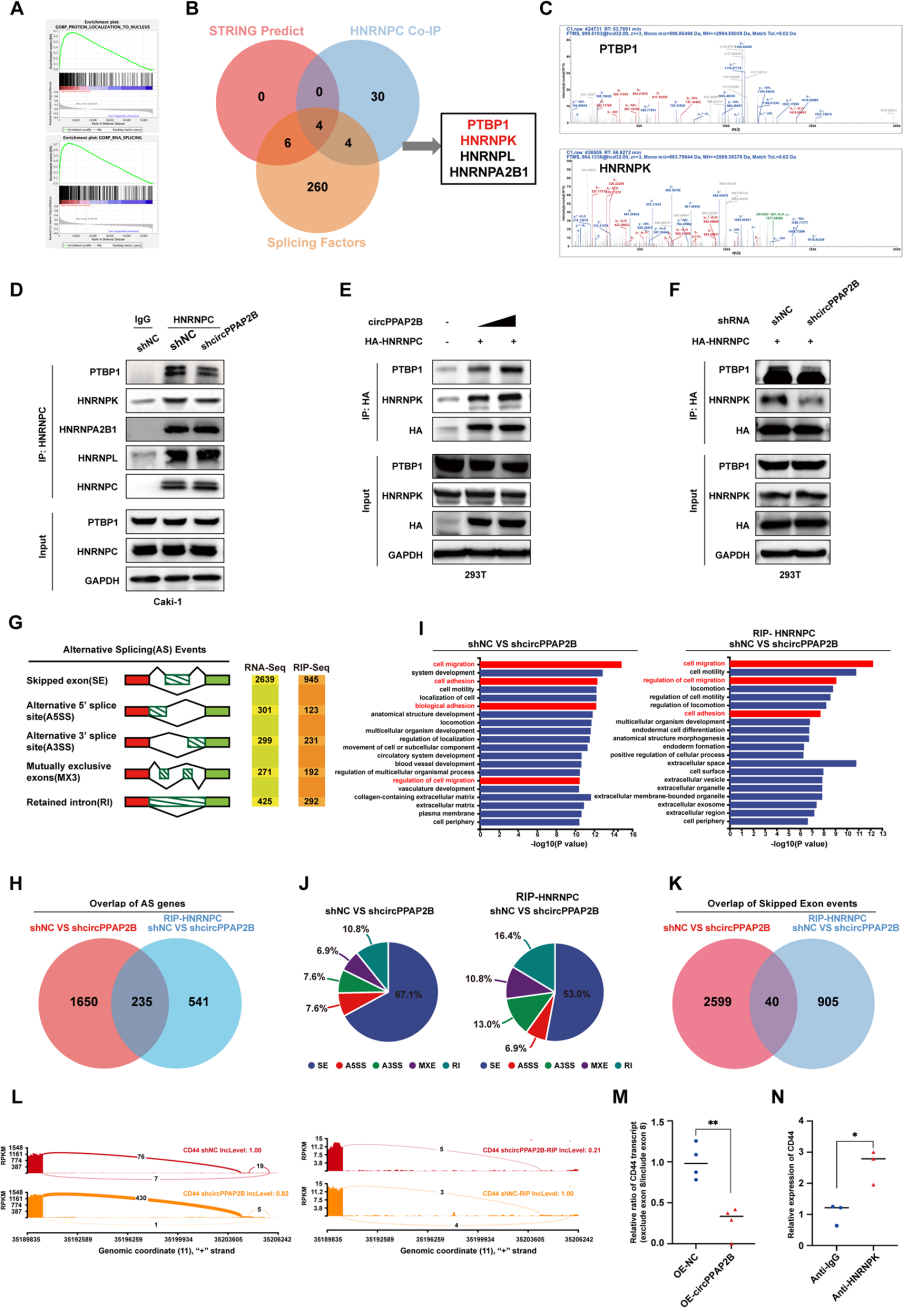
CircPPAP2B modulates the interaction between HNRNPC and splicing factors to regulate pre-mRNA alternative splicing **A** GSEA enrichment analysis was performed to reveal the relationship between HNRNPC and RNA splicing with mRNA processing in ccRCC. **B-C** Mass Spectrometry analysis and STRING prediction were employed to identify splicing factors HNRNPC regulated in ccRCC. **D** CoIP assay was performed to confirm the direct interaction between PTBP1, HNRNPK, HNRNPL, HNRNPA2B1, and HNRNPC. **E** CoIP assay was performed to confirm whether circPPAP2B overexpression influenced the interaction between HNRNPC and PTBP1 or HNRNPK. **F** CoIP assay was performed to confirm whether circPPAP2B knockdown influenced the interaction between HNRNPC and PTBP1 or HNRNPK. **G** Alternative splicing events were identified in both circPPAP2B knockdown and anti-HNRNPC circPPAP2B knockdown ccRCC cells, including skipped exon (SE), alternative 5’ splice site (A5SS), alternative 3’ splice site (A3SS), mutually exclusive exons (MXE) and retained intron. **H** 235 alternative splicing genes were shared in both circPPAP2B knockdown and anti-HNRNPC circPPAP2B knockdown ccRCC cells. **I** GO enrichment analysis identified enriched pathways with shared alternative splicing genes. **J** The Pie diagram shows the ratio of different types of alternative splicing events regulated by circPPAP2B knockdown. **K** The Venn diagram showed 40 alternative splicing events were shared in both circPPAP2B knockdown and anti-HNRNPC circPPAP2B knockdown ccRCC cells. **L** Alternative splicing events of CD44 were identified in the anti-HNRNPC RIP-sequencing assay. **M** qPCR assay was performed to validate the selected alternative of CD44 in OE-NC and OE-circPPAP2B ccRCC cells. **N** RIP-qPCR with anti-HNRNPK antibody was performed to validate the interaction between HNRNPK and CD44 alternative splicing

To investigate the role of HNRNPC in ccRCC, we performed GSEA enrichment analysis and found that RNA splicing and mRNA processing were enriched in ccRCC tissues with HNRNPC high-expression (Fig. 5A). Since HNRNPC can regulate pre-mRNA alternative splicing, we hypothesized that circPPAP2B mediated metastasis in ccRCC may occur through HNRNPC-dependent alternative splicing. To this end, we employed Mass Spectrometry analysis and STRING prediction to identify splicing factors regulated by HNRNPC in ccRCC [33]. By taking the intersection part of the Mass Spectrometry analysis result and STRING prediction identified 4 splicing factors (PTBP1, HNRNPK, HNRNPL, and HNRNPA2B1) selected for further investigation (Fig. 5B-C). Using CoIP assay we found that PTBP1, HNRNPK, HNRNPL, and HNRNPA2B1 could interact with HNRNPC, however, only interaction between PTBP1, or HNRNPK, and HNRNPC was reduced following circPPAP2B knockdown (Fig. 5D). In addition, exogenous CoIP assay further confirmed that the interaction between PTBP1, HNRNPK, and HNRNPC was regulated by circPPAP2B, suggesting that HNRNPC recruited PTPB1 and HNRNPK possibly to regulate pre-mRNA alternative splicing (Fig. 5E-F).

To test this hypothesis, we performed high-throughput RNA-sequencing analysis using RNA isolated from control cell and circPPAP2B knockdown ccRCC cells, as well as RNA isolated from anti-HNRNPC RIP assay of control cell and circPPAP2B knockdown ccRCC cells (Supplementary Table 4 and 6). As expected, we identified a large number of alternative splicing events in both RNA-seq and RIP-seq, including skipped exon (SE), alternative 5’ splice site (A5SS), alternative 3’ splice site (A3SS), mutually exclusive exons (MXE) and retained intron (RI) (Fig. 5G). By taking the intersection part, 235 alternative splicing genes were shared in both RNA-seq and RIP-seq (Fig. 5H). GO enrichment analysis revealed these shared alternative splicing genes were enriched in cell migration, cell adhesion, and regulation of cell migration, suggesting that HNRNPC-dependent alternative splicing promotes metastasis in ccRCC (Fig. 5I). Importantly, we found that SE events were the most predominant type affected by circPPAP2B (Fig. 5J). By taking the intersection part of SE events, 40 SE events were shared in RNA-seq and RIP-seq (Fig. 5K). One of the SE events *CD44*, a gene encoding a critical cell-surface glycoprotein that contributed to cell adhesin and metastasis, was selected for further validation (Fig. 5L). qPCR results showed that skipped exon events (skipping exon 8) of CD44 significantly decreased in circPPAP2B overexpression when compared to control samples (Fig. 5M). Furthermore, RIP-qPCR with anti-HNRNPK, one splicing factor recruited by HNRNPC, demonstrated that CD44 pre-mRNA significantly interacted with HNRNPK when compared to IgG control (Fig. 5N). Taken together, our results demonstrated that HNRNPC recruits PTBP1 and HNRNPK and regulates pre-mRNA alternative splicing of CD44 in ccRCC.

### CircPPAP2B directly binds to miR-182-5p

**Fig. 6:**
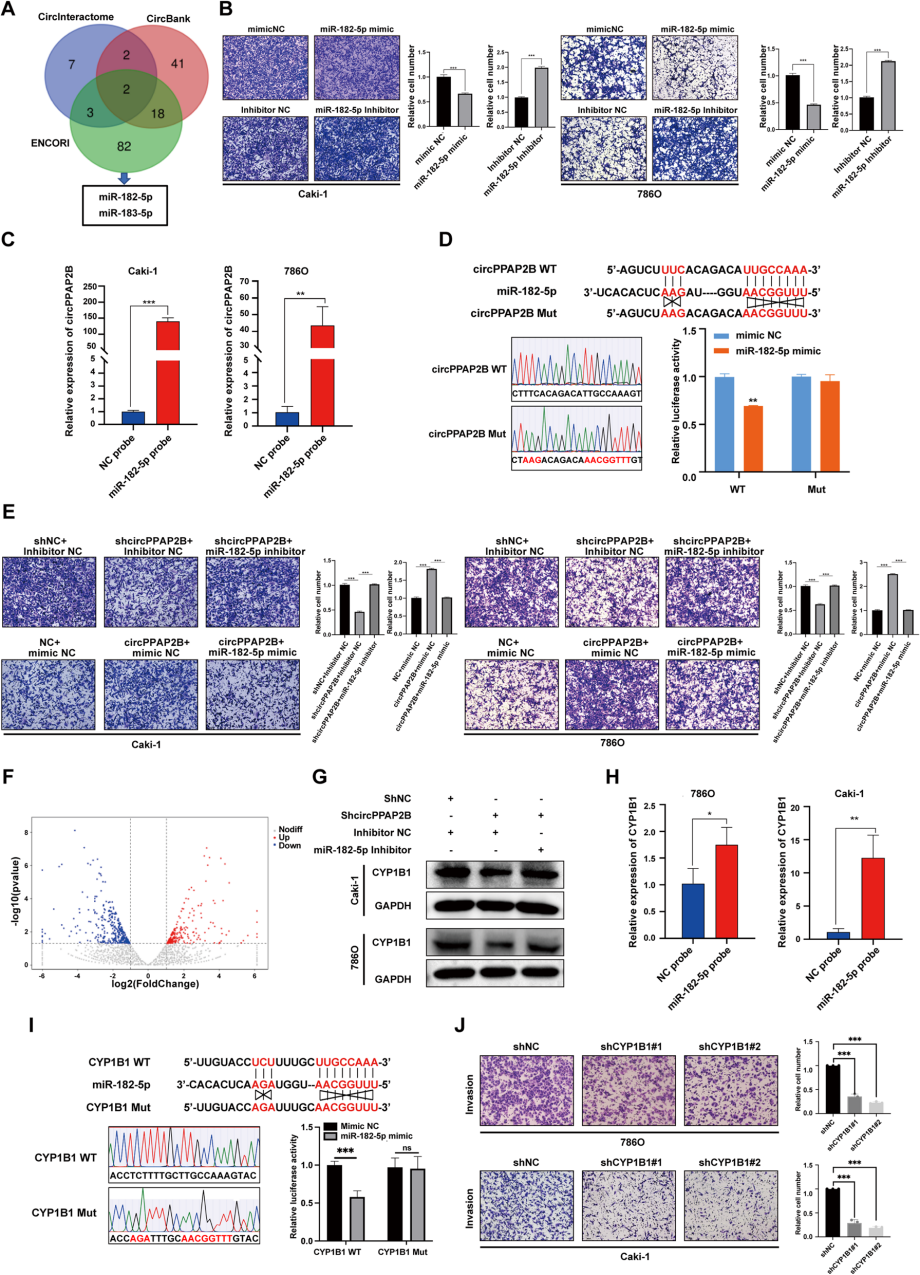
CircPPAP2B functions as a ceRNA to directly bind to miR-182-5p and upregulated CYP1B1 **A** Bioinformatics tools (circBank, ENCORI, and CircInteractome) were used to identify candidate miRNAs that potentially interact with circPPAP2B. **B** Transwell assays were performed to explore the role of miR-182-5p mimic or miR-182-5p inhibitor on the invasive ability of ccRCC cells. **C** Biotin-labeled RNA pulldown assays were performed to confirm the direct interaction between circPPAP2B and miR-182-5p. **D** Dual-luciferase reporter assay was performed to validate the direct binding and binding site between circPPAP2B and miR-182-5p. **E** Transwell assays were performed to explore the role of miR-182-5p mimic or miR-182-5p inhibitor on the circPPAP2B-mediated invasion of ccRCC cells. **F** High-throughput RNA sequencing to analyze the RNA regulated by circPPAP2B in ccRCC cells. **G** Western blotting assays to investigate the effect of miR-182-5p on circPPAP2B-mediated CYP1B1 expression. **H** Biotin-labeled RNA pulldown assay was performed to validate the direct binding between miR-182-5p and CYP1B1 mRNA.** I** Dual-luciferase reporter assay was performed to validate the direct binding and binding site between miR-182-5p and 3’UTR of CYP1B1 mRNA. **J** Transwell assay was performed to explore the role of CYP1B1 in the invasion of ccRCC cells. Data are represented as mean ± SEM. *P < 0.05, **P < 0.01, ***P < 0.001 vs. WT group

Based on our previous studies that m6A modification site mutation did not fully reverse the pro-metastatic phenotype, we hypothesized that circPPAP2B may promote ccRCC metastasis through additional mechanisms beyond interaction with RBPs. Numerous studies have shown that circRNAs can serve as miRNA sponges and antagonize miRNA-mediated post-transcriptional regulation. To investigate whether circPPAP2B functions as a miRNA sponge in ccRCC, we investigated interaction between circPPAP2B and AGO2, a component of the miRNA-induced silencing complex (miRISC) by RIP assays. Our studies revealed a significant enrichment of circPPAP2B in the anti-AGO2 group, suggesting that circPPAP2B binding may compete with specific miRNAs (Figure S6A). To identify candidate miRNAs that potentially interact with circPPAP2B, we screened through bioinformatics tools such as circBank, ENCORI, and CircInteractome [34–36]. Two miRNAs, miR-182-5p and miR-183-5p, were predicted to have complementary sequences that could potentially bind to circPPAP2B (Fig. 6A). Several observations suggest an important role for miR-182-5p. First, miR-182-5p expression was significantly decreased in ccRCC tissues when compared with normal tissues. (Figure S6B). Second, colony formation assays, CCK8 assays, and Edu assays revealed that miR-182-5p significantly inhibited proliferation in ccRCC cells (Figure S6C-E). Third, transwell assays revealed that miR-182-5p mimic inhibited the invasiveness, while miR-182-5p inhibitor promoted the invasiveness ability of ccRCC cell (Fig. 6B). We then performed biotin-labeled RNA pulldown assays to confirm interactions between circPPAP2B and miR-182-5p. The results demonstrated a substantial enrichment of circPPAP2B with a biotin-labeled miR-182-5p probe compared to the negative control (Fig. 6C). To further validate and map the binding site between circPPAP2B and miR-182-5p, we used a bioinformatics tool and constructed wild-type and mutant dual-luciferase reporter plasmid. The dual-luciferase reporter assay demonstrated that miR-182-5p mimic could significantly reduce the relative luciferase activity of wild-type dual-luciferase reporter plasmid, while it did not affect the relative luciferase activity of mutant dual-luciferase reporter plasmid (Fig. 6D). Moreover, transwell assays confirmed that miR-182-5p inhibitor could attenuate the inhibitory effect of circPPAP2B knockdown on the invasive ability of ccRCC cells. Consistently, miR-182-5p mimic could also attenuate the positive effect of circPPAP2B overexpression on the invasive ability of ccRCC cells (Fig. 6E). These results demonstrated that circPPAP2B binds to miR-182-5p to promote the invasive ability of ccRCC.

### CircPPAP2B sponges miR-182-5p results in upregulation of CYP1B1 expression in ccRCC

To explore the downstream targets of the circPPAP2B/miR-182-5p axis, we performed genome-wide next-generation RNA-sequencing (Fig. 6F). In total, 345 genes were significantly downregulated in circPPAP2B knockdown cells (|log2FC|> 1, *P*-value < 0.05). 1414 genes and 1907 genes were significantly upregulated in Caki-HI and 7860-HI, respectively. We used the bioinformatic tool Targetscan to identify the potential targets of miR-182-5p [37] (Supplementary Table 6). Interestingly, two of the miR-182-5p-regulated genes, CYP1B1 and LIMCH1, were also present in both circPPAP2B-regulated genes and HI-regulated genes identified by global RNA-seq (Figure S7A). GSEA analysis suggested that CYP1B1 was associated with the hallmarks of cell adhesion in ccRCC (Figure S7B). Kaplan–Meier survival analysis revealed that ccRCC patients with high-CYP1B1-expression have poorer overall survival than ccRCC patients with low-CYP1B1-expression (Figure S7C). Furthermore, statistical analysis showed that CYP1B1 expression was significantly associated with tumor T stage, N stage, M stage, and tumor grade (Figure S7D-SH). qPCR and Western assays revealed that circPPAP2B knockdown meaningfully reduced the mRNA and protein expression of CYP1B1, and circPPAP2B overexpression significantly increased the mRNA and protein expression of CYP1B1 (Figure S7I-K). Consistent with these results miR-182-5p inhibitor could attenuate the effect of circPPAP2B knockdown on CYP1B1 expression (Fig. 6G). We next performed biotin-labeled RNA pulldown assays to show the binding between miR-182-5p and CYP1B1 mRNA. The results from these studies showed that CYP1B1 mRNA was abundantly enriched with a biotin-labeled miR-182-5p probe when compared with negative control, suggesting the posttranscriptional regulation of miR-182-5p on CYP1B1 (Fig. 6H). To further validate and map the binding site between miR-182-5p and 3’UTR of CYP1B1 mRNA we predicted the binding site using a bioinformatics tool and constructed wild-type and mutant dual-luciferase reporter plasmid. Transient transfection experiments using dual-luciferase reporter assay showed that miR-182-5p mimic could significantly reduce the relative luciferase activity of wild-type dual-luciferase reporter plasmid, while no significant effects were detected in the relative luciferase activity of the mutated dual-luciferase reporter plasmid (Fig. 6I). Moreover, we found that CYP1B1 knockdown significantly reduced the invasive ability of ccRCC cells in transwell assays (Fig. 6J). Taken together, these results demonstrated that circPPAP2B promotes ccRCC invasion through the miR-182-5p/CYP1B1 axis.

## Discussion

CircRNAs have emerged as a novel class of single-stranded noncoding RNAs generated through pre-mRNA back-splicing or skipping events [38, 39]. The emergence of high-throughput RNA sequencing and advanced bioinformatics algorithms has led to the discovery of a vast array of circRNAs, facilitating substantial progress in the investigation of circRNAs in the context of cancer. [40, 41]. In this study, we found a novel circRNA, namely circPPAP2B, upregulated in highly invasive ccRCC cells and metastatic ccRCC tissues, promoting proliferation and metastasis in ccRCC. Here, we found that circPPAP2B interacts with HNRNPC in an m6A-dependent manner to facilitate HNRNPC nuclear translocation via stabilizing HNRNPC/Vimentin/Importin α7 ternary complex and regulates HNRNPC-dependent alternative splicing. We also report that circPPAP2B functions as a ceRNA to mediate the miR-182-5p/CYP1B1 axis in ccRCC.

Metastasis is a primary contributor to the mortality of ccRCC patients, and at present, there is a lack of effective detection methods and therapeutic approaches for addressing metastatic ccRCC [42]. To enhance our understanding of the fundamental mechanism propelling ccRCC metastasis, we created highly invasive and poorly invasive ccRCC cell lines. Subsequently, we conducted RNA sequencing to pinpoint circRNAs that exhibited differential expression. Using this approach, we discovered that circPPAP2B is associated with the invasive phenotype and metastasis potential of ccRCC. Timely detection of metastatic ccRCC can provide valuable guidance for clinical interventions [3]. Our findings indicate that circPPAP2B holds significant promise as a diagnostic tool for distinguishing between ccRCC cases with and without distant metastasis, with an impressive AUC value of 0.94. Additionally, functional investigations employing both loss-of-function and gain-of-function experiments conclusively illustrated that circPPAP2B actively facilitated the proliferation and metastasis of ccRCC. Considering the multifaceted roles of circRNAs such as miRNA sponges, RBP interacting RNAs, and protein translation templates, we investigated potential mechanisms of action. We found that circPPAP2B interacts with HNRNPC, a member of the heterogeneous nuclear ribonucleoproteins family. Although HNRNPC has been identified as an m6A reader in cancers, its regulatory mechanisms in ccRCC metastasis are poorly understood [43]. Our studies demonstrated an interaction between circPPAP2B and the acidic region of HNRNPC in an m6A-dependent manner resulting in an enrichment of m6A modification in circPPAP2B. It has been previously demonstrated that interaction between circRNA and RBPs can lead to nuclear translocation of RBPs [44]. In ccRCC cells, HNRNPC primarily resides within the nucleus, yet the precise regulatory mechanism regulating its nucleocytoplasmic translocation remains unclear. Our findings revealed that circPPAP2B plays a pivotal role in facilitating the nuclear translocation of HNRNPC by modulating HNRNPC's nondegradable ubiquitination and stabilizing the HNRNPC/Vimentin/Importin α7 ternary complex. Moreover, the m6A modification in circPPAP2B affected the relative binding affinity of HNRNPC with TRIM25 and USP10, consequently influencing the ubiquitination of HNRNPC.

Alternative splicing is a fundamental mechanism for increasing gene expression diversity and aberrant alternative splicing is significantly associated with cancer metastasis [45]. Several studies have confirmed that HNRNPC acts as a regulator of pre-mRNA alternative splicing [46]. We hypothesized that circPPAP2B mediated HNRNPC nuclear translocation, thereby influencing the regulation of alternative splicing. Indeed, studies presented here revealed that HNRNPC recruits splicing factors, PTBP1 and HNRNPK, to regulate pre-mRNA alternative splicing events in ccRCC, including the alternative splicing of CD44, a critical cell-surface glycoprotein involved in cell adhesion and metastasis.

Furthermore, our results demonstrated that the mutation of the m6A modification site in circPPAP2B did not entirely revert the pro-metastatic characteristics. This implies that circPPAP2B promotes metastasis in ccRCC through additional mechanisms distinct from its interaction with HNRNPC. Because bioinformatic tools predicted that circPPAP2B lacks translational potential, we further investigated whether circPPAP2B could function as a ceRNA in ccRCC and identified miR-182-5p as a potential binding partner. Additional experiments, such as biotin-labeled miRNA pulldown assays and dual-luciferase reporter assays, confirmed the direct binding between circPPAP2B and miR-182-5p. Rescue assays demonstrated that miR-182-5p inhibitor could attenuate the inhibition effect of circPPAP2B knockdown on invasive ability of ccRCC cells, and miR-182-5p mimic could attenuate the stimulatory effect of circPPAP2B overexpression on invasive ability of ccRCC cells. To identify potential downstream targets of the circPPAP2B/miR-182-5p axis, we next conducted RNA sequencing and bioinformatic TargetScan analysis. Our results indicated that CYP1B1 represented a bonafide target of the circPPAP2B/miR-182-5p axis. Previous studies have linked CYP1B1 to the progression of various cancers. In our study, we observed a significant association between CYP1B1 expression and tumor M stage, as well as poor prognosis in ccRCC patients. Biotin-labeled RNA pulldown assays and dual-luciferase reporter assays confirmed the direct binding between miR-182-5p and CYP1B1 mRNA. Moreover, CYP1B1 knockdown significantly reduced the invasive ability of ccRCC cells.

## Conclusion

**Fig. 7:**
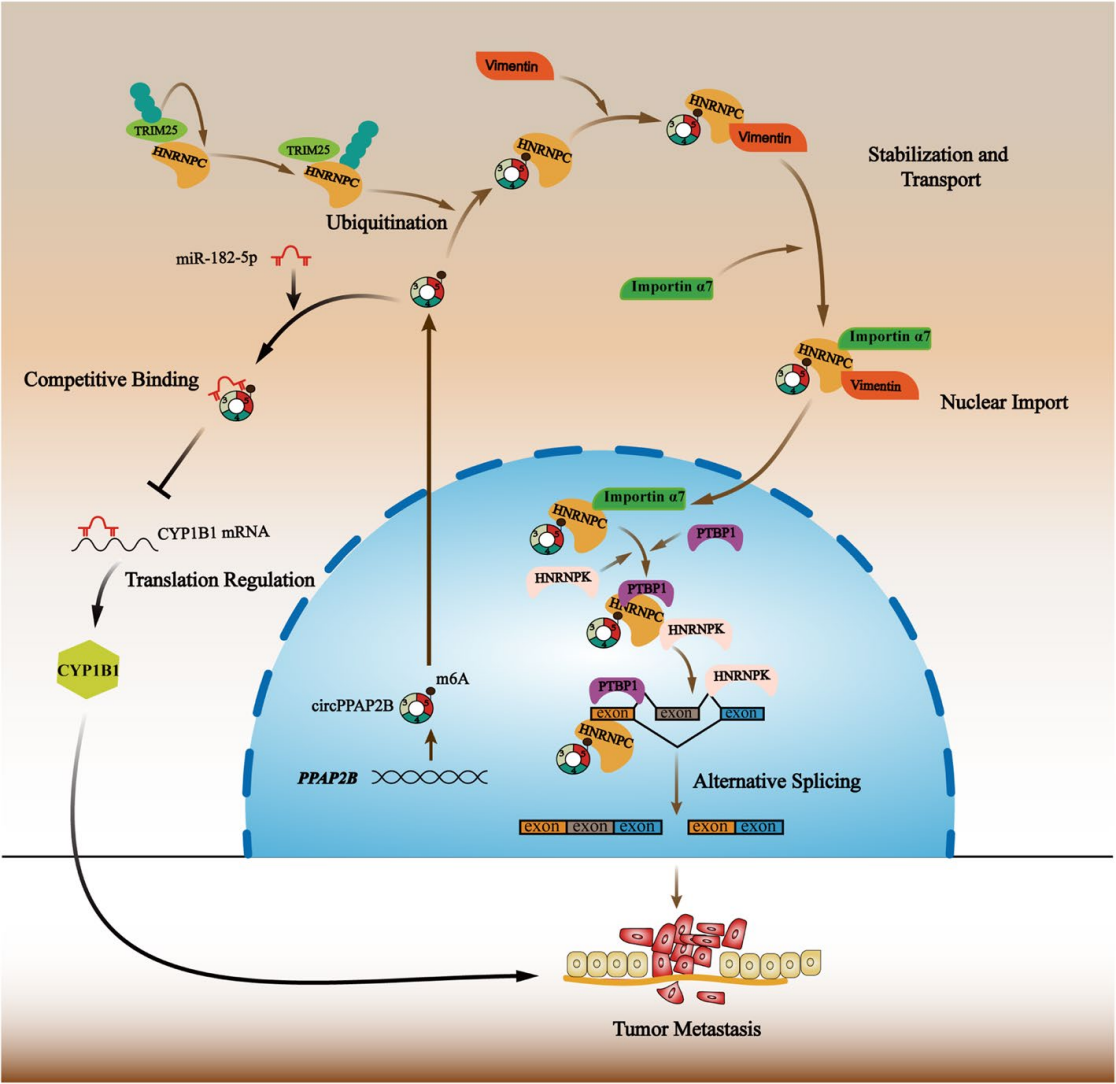
Schematic representation of the possible mechanism of circPPAP2B in ccRCC metastasis CircPPAP2B is derived from exons 3 to 5 of the *PPAP2B* gene through backsplicing in the nucleus. CircPPAP2B not only interacts with HNRNPC in an m6A-dependent manner to facilitate HNRNPC nuclear translocation via stabilizing HNRNPC/Vimentin/Importin α7 ternary complex, but also functions as a miRNA sponge to directly bind to miR-182-5p and modulate CYP1B1 expression, thereby promoting cancer metastasis in ccRCC

In summary, our study reveals that circPPAP2B plays a significant role in ccRCC metastasis. The upregulation of circPPAP2B expression levels drives metastasis through several pathways (Fig. 7). To begin with, circPPAP2B engages in a direct interaction with HNRNPC in an m6A-dependent manner. This interaction results in the promotion of HNRNPC's nuclear translocation by orchestrating nondegradable ubiquitination of HNRNPC and reinforcing the stability of the HNRNPC/Vimentin/Importin α7 ternary complex. Additionally, circPPAP2B recruits splicing factors, PTBP1 and HNPNPK, to oversee the regulation of pre-mRNA alternative splicing. Secondly, circPPAP2B serves as a miRNA sponge, forming a direct bond with miR-182-5p and regulating CYP1B1 expression in ccRCC. Our discoveries offer a comprehensive comprehension of the intricate mechanistic pathway orchestrated by circPPAP2B, encompassing HNRNPC-dependent alternative splicing and the miR-182-5p/CYP1B1 axis. These insights underscore the potential of circPPAP2B as a promising therapeutic target for managing metastasis in ccRCC.

### Supplementary Information

**Additional file 1: Figure S1. **High-throughput RNA sequencing and related analysis in highly and poorly invasive ccRCC cells. A-B) High-throughput RNA sequencing to analyze the expression profiles of mRNAs in highly and poorly invasive ccRCC cells Caki-1 and 786O. C-D) GO enrichment analysis to analyze enriched biological processes with differentially expressed genes between highly invasive and poorly invasive ccRCC cells Caki-1 and 786O. E-F) KEGG pathway analysis to analyze enriched pathways with differentially expressed genes between highly invasive and poorly invasive ccRCC cells Caki-1 and 786O.

**Additional file 2: Figure S2. **CircPPAP2B is overexpressed in ccRCC and correlates with metastasis. A) circPPAP2B expression in ccRCC cells (Caki-1, 786O, ACHN and Caki-2) and HK-2. B) RNA FISH was performed to detect circPPAP2B expression in highly and poorly invasive ccRCC cells Caki-1 and 786O. C) qPCR was performed to detect circPPAP2B expression in highly and poorly invasive ccRCC cells Caki-1 and 786O. D) Receiver operating characteristic analysis was performed to evaluate the diagnostic value of circPPAP2B in differentiating between ccRCC with and without distant metastasis.

**Additional file 3: Figure S3.** CircPPAP2B knockdown and overexpression efficiency in ccRCC cells. A) Quantification of circPPAP2B expression assessed by qPCR in ccRCC cell lines treated with siRNA-targeted circPPAP2B or negative control. B) Quantification of circPPAP2B expression assessed by qPCR in ccRCC cell lines treated with OE-NC or OE-circPPAP2B. C) Representative images of ccRCC cells transfected with shNC or shcircPPAP2B. D) Bioinformatics tool ORFinder to predict ORFs in circPPAP2B sequence. Data are represented as mean ± SEM. ***P <0.001 vs. WT group.

**Additional file 4: Figure S4. **CircPPAP2B regulates HNRNPC nuclear translocation via stabilizing HNRNPC/Vimentin/Importin α7 interaction. A) RNA FISH was performed to determine the role of circPPAP2B and its m6A modification site on the subcellular localization of HNRNPC in ccRCC cell lines. B) Mass Spectrometry analysis revealed the interaction between HNRNPC and Vimentin. C) CoIP assay was performed to confirm the direct interaction between HNRNPC and Importin α7. D) The predicted 3D model of HNRNPC/Vimentin/Importin α7 ternary complex by HDOCK software. E-F) CoIP assay was performed to determine the role of circPPAP2B on the interaction between HNRNPC and Importin α7 in both 293T and Caki-1 cells. G) CoIP assay was performed to identify the specific domain of HNRNPC which interacts with Importin α7.

**Additional file 5: Figure S5. **CircPPAP2B regulates HNRNPC nondegradable ubiquitination via TRIM25 and USP10. A) Protein half-life assays were performed to explore the role of circPPAP2B on HNRNPC expression. B) Western blotting assays were performed to explore the role of circPPAP2B knockdown or overexpression on HNRNPC expression. C) CoIP assay was performed to explore whether circPPAP2B regulates the ubiquitination of HNRNPC in 293T cells. D) CoIP assay was performed to determine the role of circPPAP2B on the interaction between HNRNPC and USP10. E) The predicted 3D model of HNRNPC/TRIM25/USP10 by HDOCK software. F) CoIP assay was performed to explore whether circPPAP2B regulates the interaction between HNRNPC and TRIM25 or USP10. G) CoIP assay was performed to determine the role of TRIM25 on ubiquitination levels of HNRNPC. H) CoIP assay was performed to determine the role of TRIM25 on the interaction of HNRNPC with Importin α7.

**Additional file 6: Figure S6.** miR-182-5p is downregulated in ccRCC and inhibits the proliferation of ccRCC cells. A) RIP assays were performed to explore the interaction between circPPAP2B and AGO2. B) miR-183-5p expression in ccRCC tissues (n=521) and normal kidney tissues (n=71) in the TCGA database. C) The colony formation assays were performed to the role of miR-182-5p mimic and inhibitor on the proliferation of ccRCC cells. D) CCK8 assays were performed to role of miR-182-5p mimic and inhibitor on the proliferation of ccRCC cells. E) EdU assays were performed to role of miR-182-5p mimic and inhibitor on the proliferation of ccRCC cells. Data are represented as mean ± SEM. *P <0.05, **P< 0.01, ***P<0.001 vs. WT group.

**Additional file 7: Figure S7.** CircPPAP2B upregulates CYP1B1 expression in ccRCC. A) Intersection analysis of RNA sequencing data and bioinformatic tools to identify the potential targets of miR-182-5p. B) GSEA enrichment analysis was performed to reveal the relationship between CYP1BA and biological processing in ccRCC. C) Kaplan-Meier survival analysis to reveal the association between CYP1B1 expression and prognosis of ccRCC patients. D-H) Statistical analysis to reveal the association between CYP1B1 expression and tumor T stage, N stage, M stage, and tumor grade. I) Western blotting assays to investigate the effect of circPPAP2B knockdown on CYP1B1 expression. J) Western blotting assays to investigate the effect of circPPAP2B overexpression on CYP1B1 expression. K) qPCR assays were performed to investigate the effect of circPPAP2B overexpression on the mRNA level of CYP1B1. Data are represented as mean ± SEM. *P < 0.05, **P < 0.01, ***P <0.001 vs. WT group.

**Additional file 8: Supplementary Methods.**

**Additional file 9: Supplementary Table 1.** Identification of circPPAP2B in ccRCC.

**Additional file 10: Supplementary Table 2.** Predicted IRESites of circPPAP2B

**Additional file 11: Supplementary Table 3.** Identification of circPPAP2B-binding proteins by Mass spectrometry analysis.

**Additional file 12: Supplementary Table 4.** Skipped exon events of RNA-Seq.

**Additional file 13: Supplementary Table 5.** Skipped exon events of RIP-Seq.

**Additional file 14: Supplementary Table 6.** Predicted miR-182-5p-binding mRNA by TargedScan.

**Additional file 15: Supplementary Table 7.** Antibodies and Primers.

## Data Availability

The data used and analyzed in this article were included within the article or the additional files. Please contact the corresponding author for data requests.

## Abbreviations

RCC: Renal cell carcinoma
circRNAs: Circular RNAs
ccRCC: Clear cell renal cell carcinoma
MeRIP: RNA methylation immunoprecipitation
RIP: RNA immunoprecipitation
CoIP: Co-immunoprecipitation
Pre-mRNA: Precursor mRNA
HNRNPC: Heterogeneous nuclear ribonucleoprotein C
HI: Highly invasive
PI: Poorly invasive
GO: Gene ontology
qPCR: Quantitative reverse transcription PCR
FISH: Fluorescence in situ hybridization
HK-2: Human Kidney-2
AUC: Area under the curve
RBPs: RNA binding proteins
ORFs: Open reading frames
IRES: Internal ribosome entry site
SDS-PAGE: Sodium Dodecyl Sulfate PolyAcrylamide Gel Electrophoresis
RIP: RNA immunoprecipitation
m6A: N6-methyladenosine
MTC: Methyltransferase complex
EMT: Epithelial-to-mesenchymal transition
DUBs: Deubiquitinases
SE: Skipped exon
A5SS: Alternative 5’ splice site
A3SS: Alternative 3’ splice site
MXE: Mutually exclusive exons
RI: Retained intron
miRISC: MiRNA-induced silencing complex
