# Supplementary figures and images for: CircPPAP2B controls metastasis of clear cell renal cell carcinoma via HNRNPC-dependent alternative splicing and targeting the miR-182-5p/CYP1B1 axis
Source: Mol Cancer. 2024 Jan 6;23:4. doi: 10.1186/s12943-023-01912-w (PMC10770969; doi:10.1186/s12943-023-01912-w)

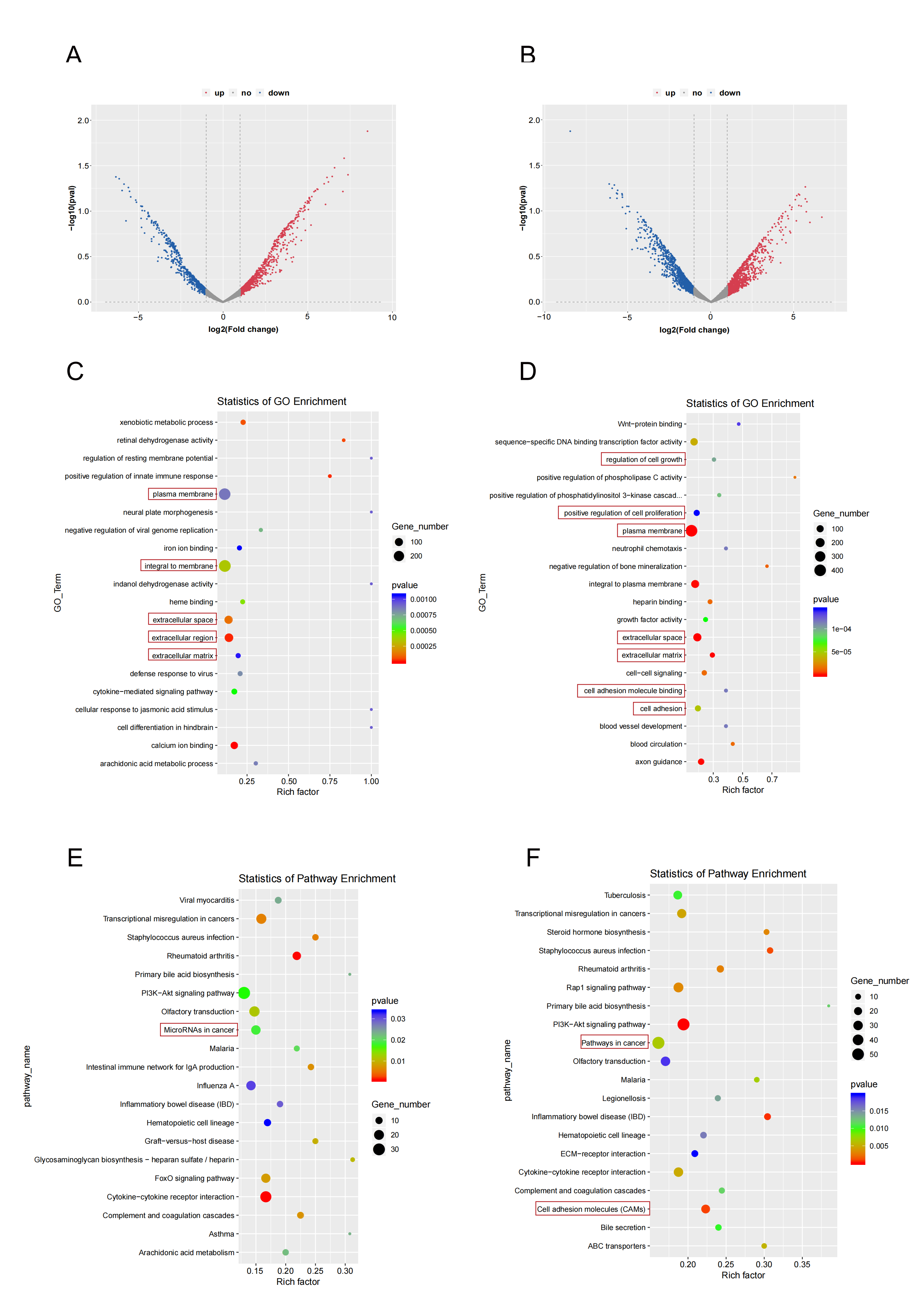

Supplement: Supplementary file 1 — Additional file 1: Figure S1. High-throughput RNA sequencing and related analysis in highly and poorly invasive ccRCC cells. A-B) High-throughput RNA sequencing to analyze the expression profiles of mRNAs in highly and poorly invasive ccRCC cells Caki-1 and 786O. C-D) GO enrichment analysis to analyze enriched biological processes with differentially expressed genes between highly invasive and poorly invasive ccRCC cells Caki-1 and 786O. E-F) KEGG pathway analysis to analyze enriched pathways with differentially expressed genes between highly invasive and poorly invasive ccRCC cells Caki-1 and 786O. [file 12943_2023_1912_MOESM1_ESM.tif]

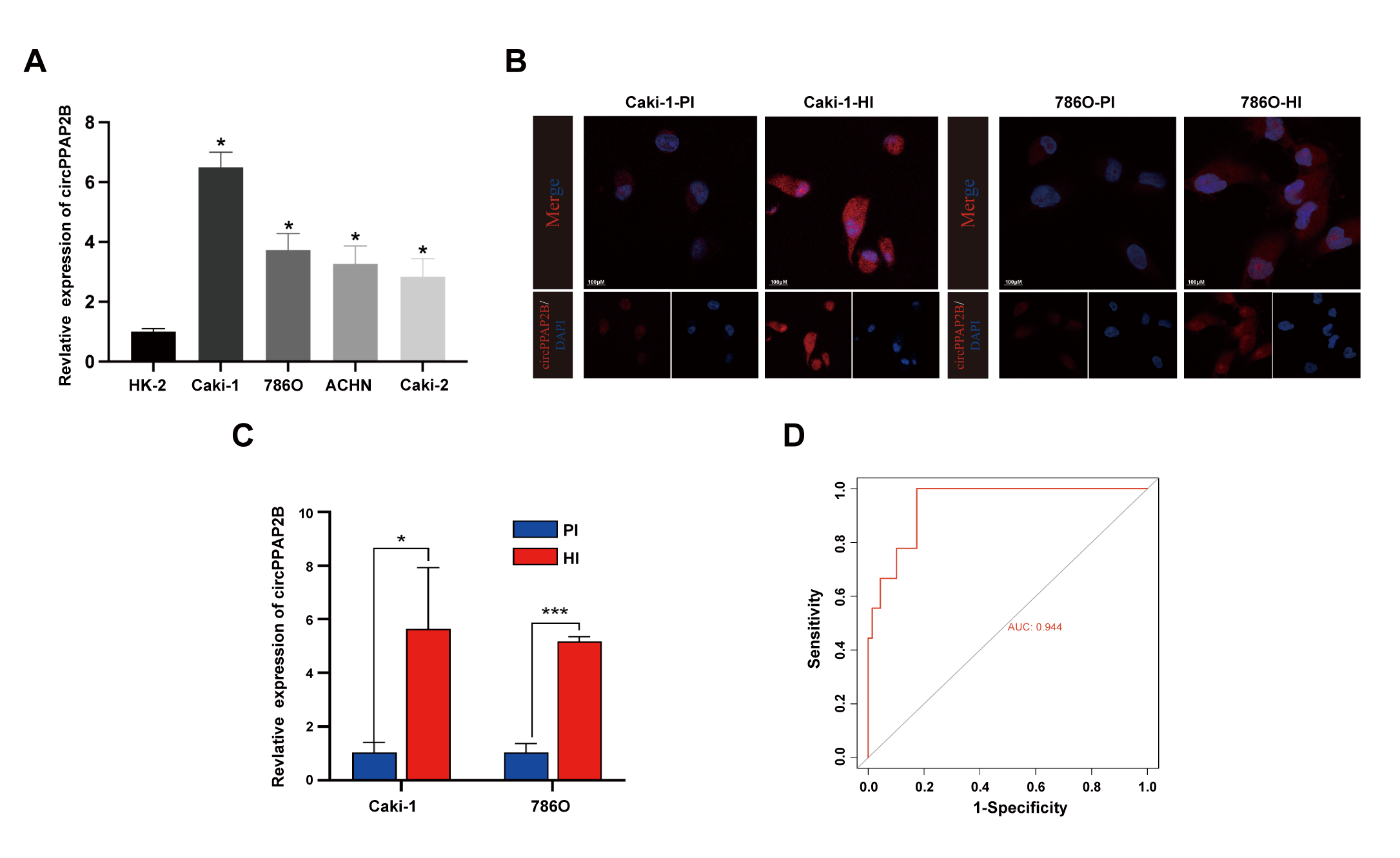

Supplement: Supplementary file 2 — Additional file 2: Figure S2. CircPPAP2B is overexpressed in ccRCC and correlates with metastasis. A) circPPAP2B expression in ccRCC cells (Caki-1, 786O, ACHN and Caki-2) and HK-2. B) RNA FISH was performed to detect circPPAP2B expression in highly and poorly invasive ccRCC cells Caki-1 and 786O. C) qPCR was performed to detect circPPAP2B expression in highly and poorly invasive ccRCC cells Caki-1 and 786O. D) Receiver operating characteristic analysis was performed to evaluate the diagnostic value of circPPAP2B in differentiating between ccRCC with and without distant metastasis. [file 12943_2023_1912_MOESM2_ESM.tif]

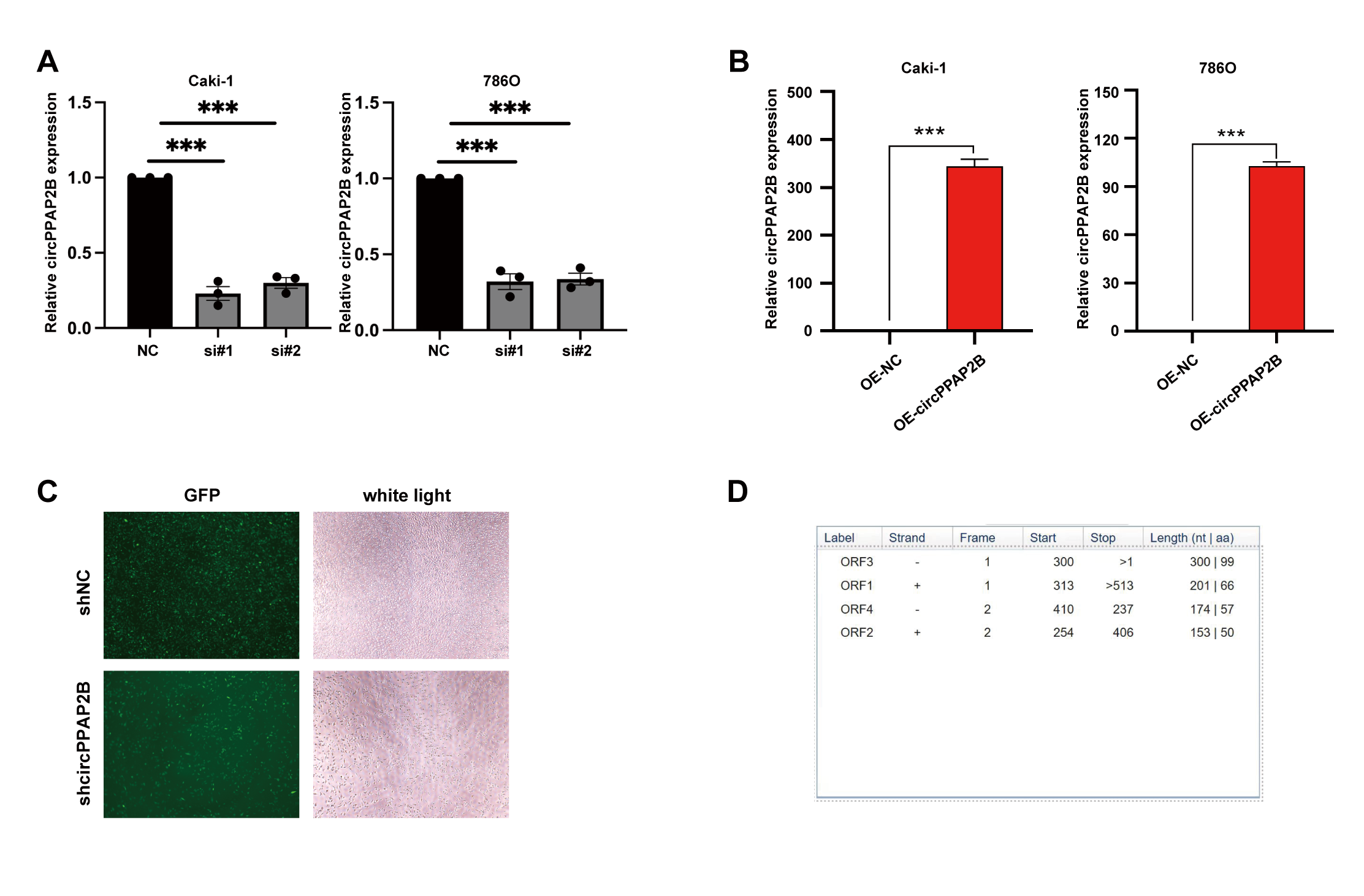

Supplement: Supplementary file 3 — Additional file 3: Figure S3. CircPPAP2B knockdown and overexpression efficiency in ccRCC cells. A) Quantification of circPPAP2B expression assessed by qPCR in ccRCC cell lines treated with siRNA-targeted circPPAP2B or negative control. B) Quantification of circPPAP2B expression assessed by qPCR in ccRCC cell lines treated with OE-NC or OE-circPPAP2B. C) Representative images of ccRCC cells transfected with shNC or shcircPPAP2B. D) Bioinformatics tool ORFinder to predict ORFs in circPPAP2B sequence. Data are represented as mean ± SEM. ***P <0.001 vs. WT group. [file 12943_2023_1912_MOESM3_ESM.tif]

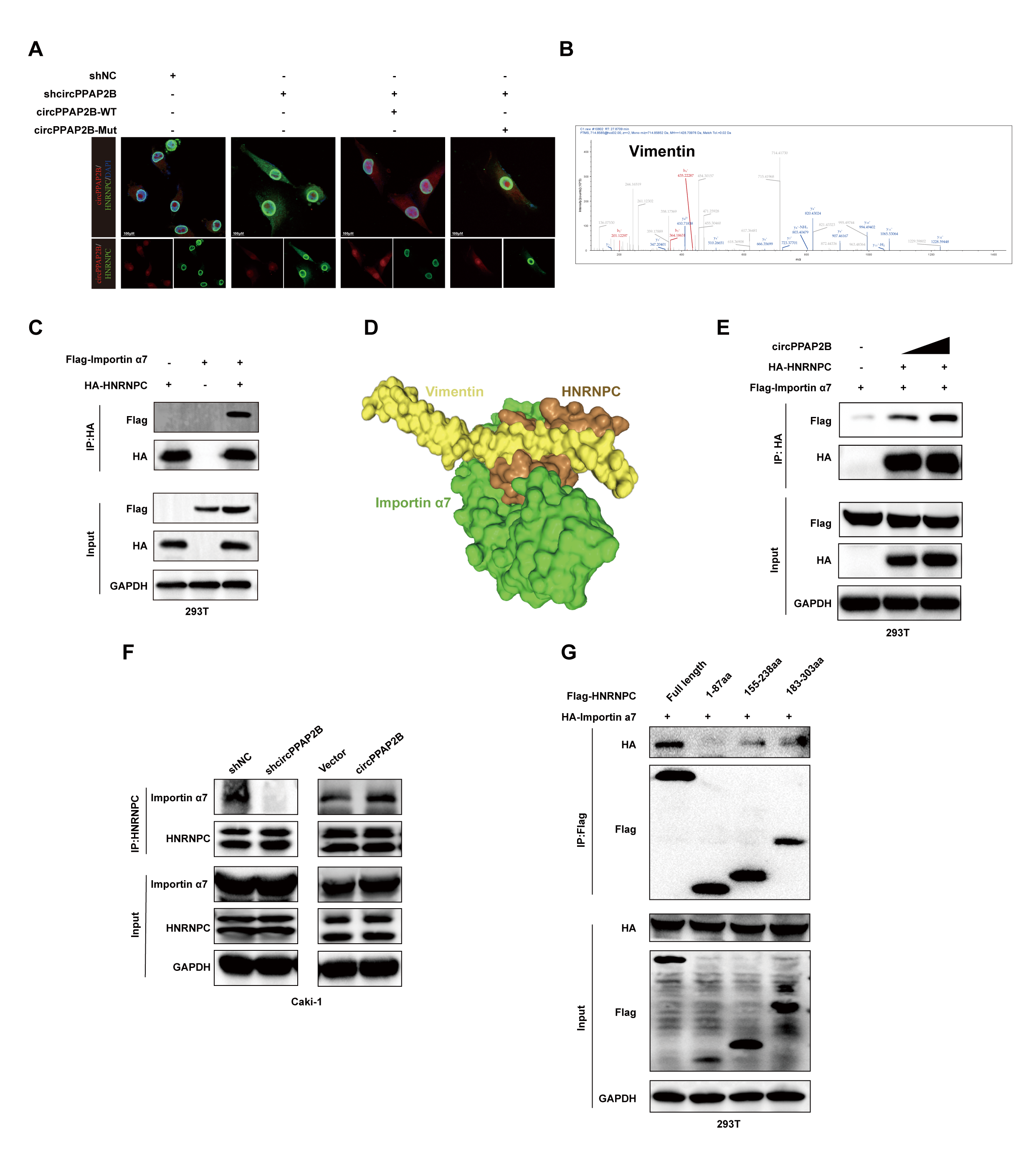

Supplement: Supplementary file 4 — Additional file 4: Figure S4. CircPPAP2B regulates HNRNPC nuclear translocation via stabilizing HNRNPC/Vimentin/Importin α7 interaction. A) RNA FISH was performed to determine the role of circPPAP2B and its m6A modification site on the subcellular localization of HNRNPC in ccRCC cell lines. B) Mass Spectrometry analysis revealed the interaction between HNRNPC and Vimentin. C) CoIP assay was performed to confirm the direct interaction between HNRNPC and Importin α7. D) The predicted 3D model of HNRNPC/Vimentin/Importin α7 ternary complex by HDOCK software. E-F) CoIP assay was performed to determine the role of circPPAP2B on the interaction between HNRNPC and Importin α7 in both 293T and Caki-1 cells. G) CoIP assay was performed to identify the specific domain of HNRNPC which interacts with Importin α7. [file 12943_2023_1912_MOESM4_ESM.tif]

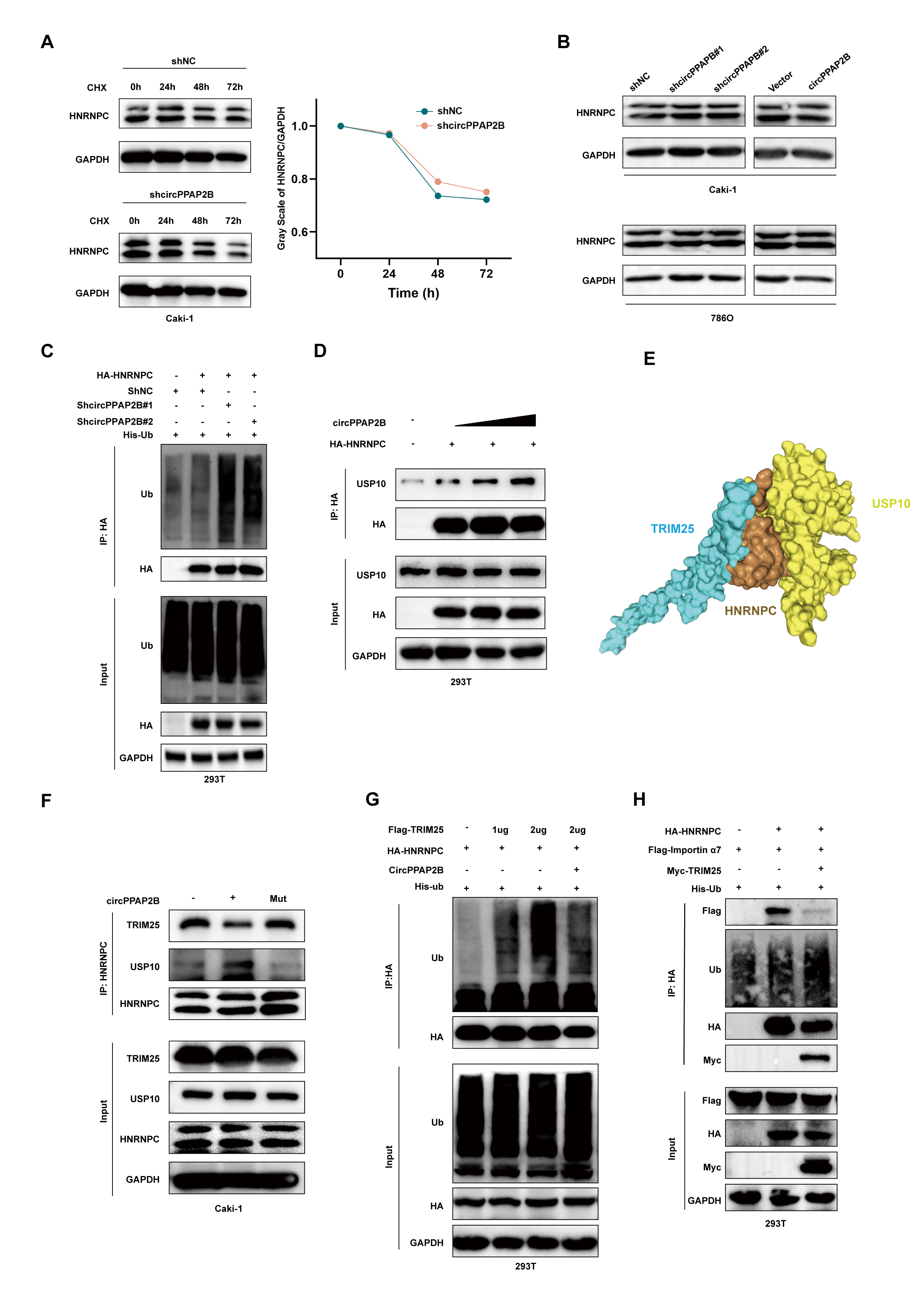

Supplement: Supplementary file 5 — Additional file 5: Figure S5. CircPPAP2B regulates HNRNPC nondegradable ubiquitination via TRIM25 and USP10. A) Protein half-life assays were performed to explore the role of circPPAP2B on HNRNPC expression. B) Western blotting assays were performed to explore the role of circPPAP2B knockdown or overexpression on HNRNPC expression. C) CoIP assay was performed to explore whether circPPAP2B regulates the ubiquitination of HNRNPC in 293T cells. D) CoIP assay was performed to determine the role of circPPAP2B on the interaction between HNRNPC and USP10. E) The predicted 3D model of HNRNPC/TRIM25/USP10 by HDOCK software. F) CoIP assay was performed to explore whether circPPAP2B regulates the interaction between HNRNPC and TRIM25 or USP10. G) CoIP assay was performed to determine the role of TRIM25 on ubiquitination levels of HNRNPC. H) CoIP assay was performed to determine the role of TRIM25 on the interaction of HNRNPC with Importin α7. [file 12943_2023_1912_MOESM5_ESM.tif]

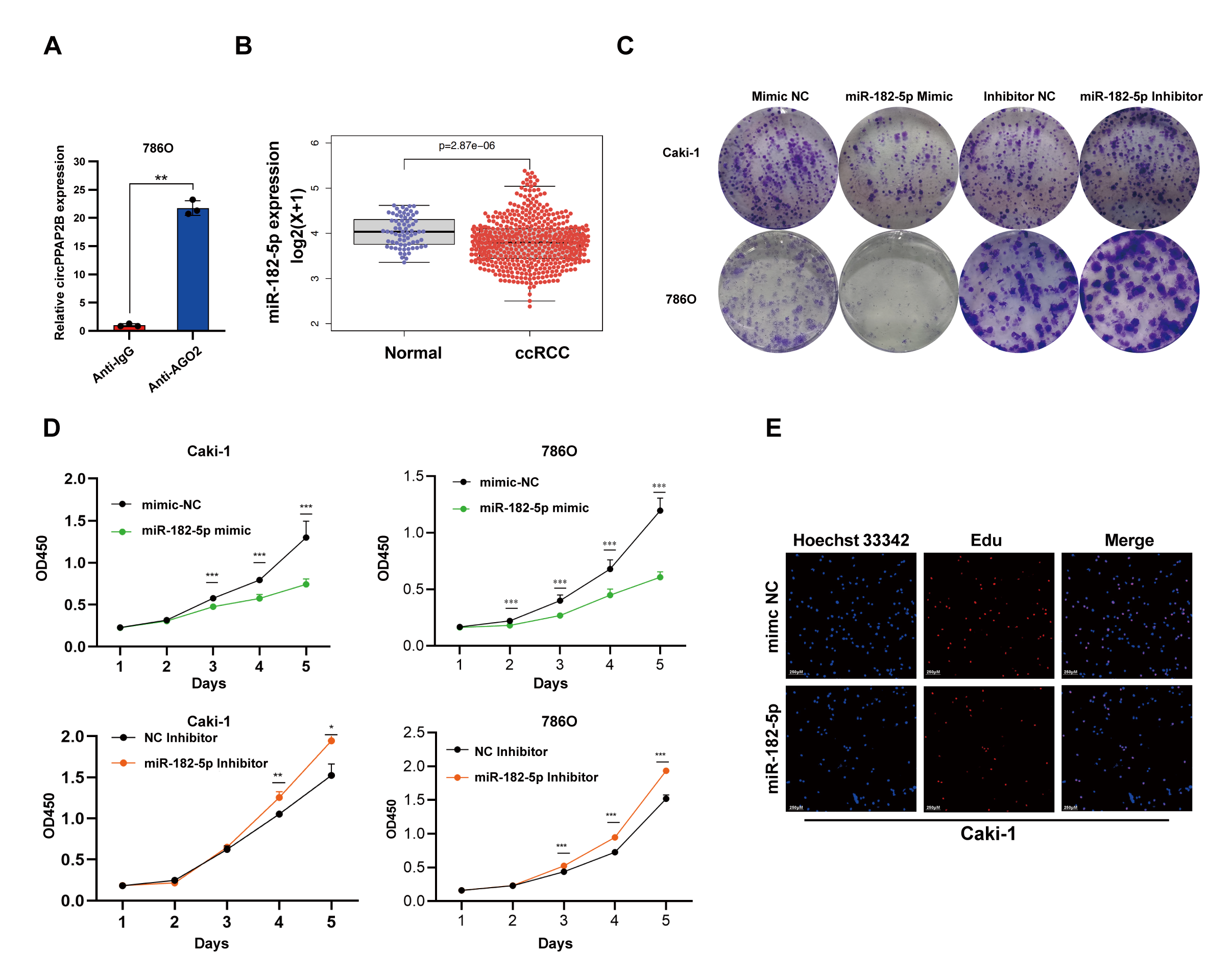

Supplement: Supplementary file 6 — Additional file 6: Figure S6. miR-182-5p is downregulated in ccRCC and inhibits the proliferation of ccRCC cells. A) RIP assays were performed to explore the interaction between circPPAP2B and AGO2. B) miR-183-5p expression in ccRCC tissues (n=521) and normal kidney tissues (n=71) in the TCGA database. C) The colony formation assays were performed to the role of miR-182-5p mimic and inhibitor on the proliferation of ccRCC cells. D) CCK8 assays were performed to role of miR-182-5p mimic and inhibitor on the proliferation of ccRCC cells. E) EdU assays were performed to role of miR-182-5p mimic and inhibitor on the proliferation of ccRCC cells. Data are represented as mean ± SEM. *P <0.05, **P< 0.01, ***P<0.001 vs. WT group. [file 12943_2023_1912_MOESM6_ESM.tif]

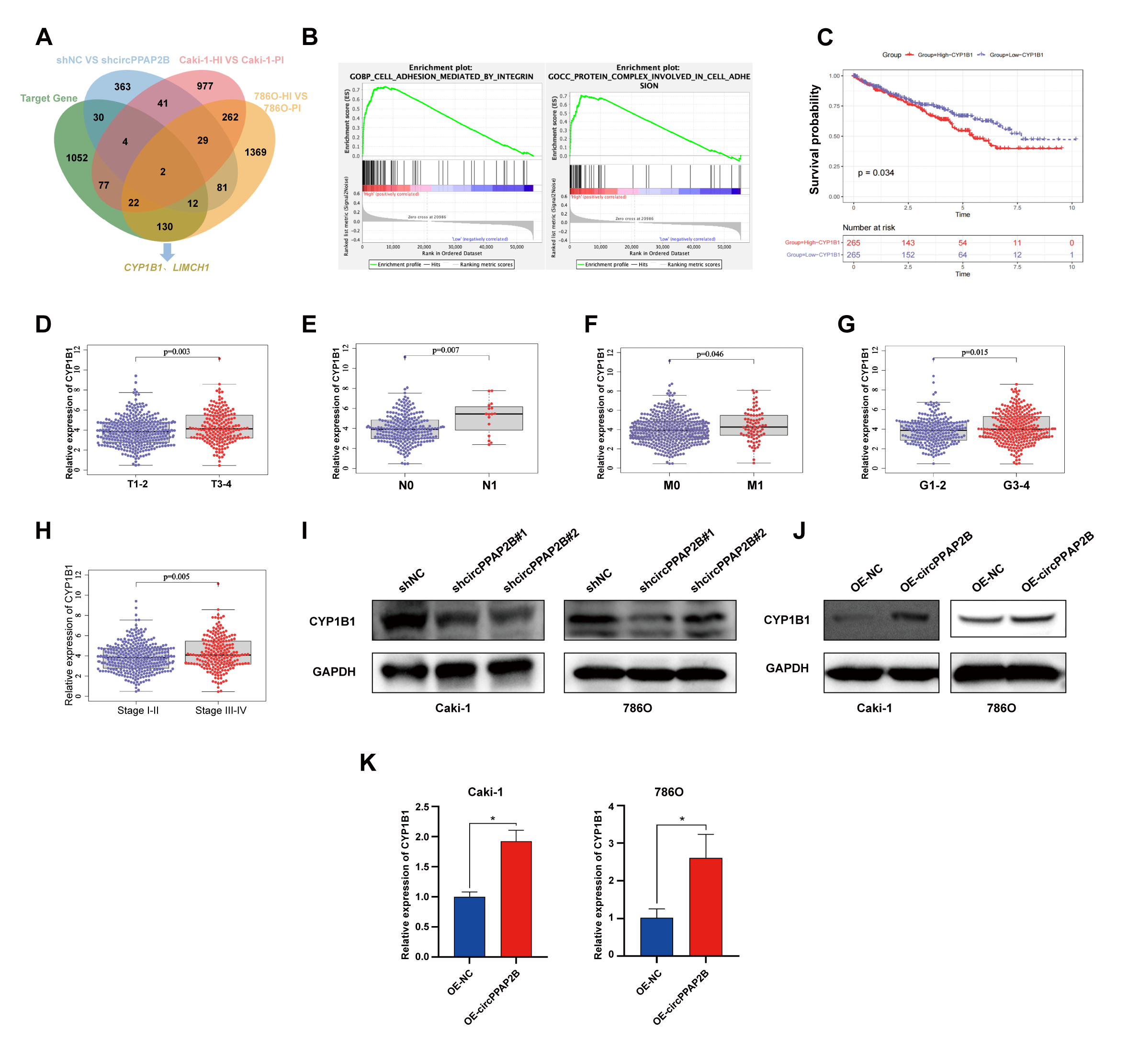

Supplement: Supplementary file 7 — Additional file 7: Figure S7. CircPPAP2B upregulates CYP1B1 expression in ccRCC. A) Intersection analysis of RNA sequencing data and bioinformatic tools to identify the potential targets of miR-182-5p. B) GSEA enrichment analysis was performed to reveal the relationship between CYP1BA and biological processing in ccRCC. C) Kaplan-Meier survival analysis to reveal the association between CYP1B1 expression and prognosis of ccRCC patients. D-H) Statistical analysis to reveal the association between CYP1B1 expression and tumor T stage, N stage, M stage, and tumor grade. I) Western blotting assays to investigate the effect of circPPAP2B knockdown on CYP1B1 expression. J) Western blotting assays to investigate the effect of circPPAP2B overexpression on CYP1B1 expression. K) qPCR assays were performed to investigate the effect of circPPAP2B overexpression on the mRNA level of CYP1B1. Data are represented as mean ± SEM. *P < 0.05, **P < 0.01, ***P <0.001 vs. WT group. [file 12943_2023_1912_MOESM7_ESM.tif]
