# Supplementary material for: CircPPAP2B controls metastasis of clear cell renal cell carcinoma via HNRNPC-dependent alternative splicing and targeting the miR-182-5p/CYP1B1 axis
Source: Mol Cancer. 2024 Jan 6;23:4. doi: 10.1186/s12943-023-01912-w (PMC10770969; doi:10.1186/s12943-023-01912-w)
Supplement: Supplementary file 8 — Additional file 8: Supplementary Methods. [file 12943_2023_1912_MOESM8_ESM.docx]

**Methods**

**Human tissue samples**

In total, 78 paired primary ccRCC tumor tissues and adjacent non-tumor tissues were collected from patients who underwent surgery at Nanfang Hospital (Guangzhou, China). All tissues were freshly frozen in dry ice or RNA Isolation Buffer (Thermo Fisher Scientific) and stored at -80℃.

**Cell culture, transfection, and lentiviral infection**

All human RCC cell lines (Caki-1, 786-O, ACHN, and Caki-2) and human embryonic kidney cell line HEK293T were obtained from the American Type Culture Collection (ATCC). Caki-1, ACHN, Caki-2, and HEK293T cells were cultured in a DMEM medium (Procell, Wuhan). 786-O cells were cultured in RPMI 1640 medium. All medium was supplemented with 10% Fetal Bovine Serum (Haixing, Suzhou) and 1% penicillin/streptomycin (NCMbio, Suzhou) for cell culture.

For the transfection of siRNAs (Tsingke, Beijing) and plasmids into RCC cell lines, we used Lipofectamine 3000 (Invitrogen) following the manufacturer's instructions. For HEK293T cells, PEI (Polysciences) was employed for transfection according to the manufacturer's protocol. All the siRNAs and plasmids were purchased from IGE Company (Guangzhou) and the sequences are shown in **Supplementary Table 7**.

To produce lentivirus, HEK293T cells were cultured in a 10cm dish until reached 70% confluence. Subsequently, the cells were cotransfected with a lentiviral vector of interest (3μg), the pMD2.G vector (2μg), and the psPAX2 vector (3μg) in 750μl Opti-MEM. After 72 hours post-transfection, viral particles were collected from the supernatant of cultured HEK293T cells by passing through a Millex-GP Filter Unit (Jetfil, Guangzhou) with a 0.45 μm pore size. For lentiviral infection, 1 ml of packaged lentivirus, supplemented with 10μg/ml of polybrene (Beyotime), was used to culture cells for 8 hours. Cells were subsequently selected with 2 μg/ml of puromycin for 7 days.

**Characterization of poorly invasive and highly invasive renal carcinoma cells**

To establish highly invasive and poorly invasive ccRCC cell lines, transwell invasion assays were employed. Specifically, Caki-1 and 786-O cells were seeded onto transwell chambers with Matrigel (Corning, NY). Subsequently, a certain incubation period allowed for cell invasion. After the designated incubation time, ccRCC cells that had invaded and passed through the transwell chamber's membrane, settling below the chamber, were considered highly invasive cells. Conversely, ccRCC cells remaining above the chamber's membrane, indicating lower invasive capacity, were categorized as poorly invasive cells. To ensure the robustness and reliability of the models, this protocol was meticulously repeated 20 times. As a result, highly invasive cell lines (Caki-1-HI and 786O-HI) and poorly invasive cell lines (Caki-1-PI and 786O-PI) were successfully identified.

**RNA fluorescence in situ hybridization (FISH)**

FISH was performed to detect the localization of circPPAP2B in Caki-1 and 786-O cells. Caki-1 and 786-O cells were fixed with 4% formaldehyde for 15 minutes to preserve the cellular structure and RNA content. After fixation, cells were permeabilized with 0.1% Triton-X 100 for 15 minutes. Hybridization was carried out in the presence of a 4 μM Cy3-conjugated circPPAP2B probe. The cells were incubated at 37℃ in the dark overnight to facilitate specific binding of the probe to the circPPAP2B. Following hybridization, cell nuclei were counterstained with 4',6-diamidino-2-phenylindole (DAPI), a fluorescent dye that binds to DNA. DAPI staining helps visualize the cellular nuclei. The cells were washed three times with phosphate-buffered saline (PBS) at room temperature (RT) for 15 minutes each. Washing removes any unbound or non-specifically bound probes, reducing the background signal. Finally, the images were acquired using an LSM980 confocal microscope (Zeiss).

**qPCR analysis**

For the quantitative analysis of mRNA and circRNA expression levels, a two-step qPCR approach was employed. HiScript II Q RT SuperMix for qPCR (+gDNA wiper) from Vazyme (Nanjing, China) was utilized to synthesize the first-strand cDNA from the total RNA samples. This process converts RNA into complementary DNA (cDNA), which serves as a template for the subsequent qPCR reaction. AceQ qPCR SYBR Green Master Mix, also from Vazyme (Nanjing, China), was employed for the qPCR analysis. SYBR Green dye binds to double-stranded DNA during the PCR process, enabling real-time monitoring of amplification. To ensure accurate normalization of the mRNA and circRNA expression levels, the housekeeping gene GAPDH was used as an internal control.

**Western blotting**

Cells were lysed with RIPA lysis buffer (Cell Signaling Technology) supplemented with a protease inhibitor cocktail (Millipore). An equal amount of protein extracts was loaded onto a sodium dodecyl sulfate-polyacrylamide gel electrophoresis (SDS-PAGE) gel. After separation, the proteins were transferred from the gel onto a polyvinylidene fluoride (PVDF) membrane from Millipore. To prevent non-specific binding, the PVDF membrane was blocked using a 5% bovine serum albumin (BSA) solution in TBST buffer. The blocked membrane was then incubated with a specific primary antibody overnight at 4℃ and a secondary antibody labeled with horseradish peroxidase (HRP). The protein bands were visualized using an imaging system called GelView 6000 Pro from BLT (China).

**Immunofluorescence and immunohistochemistry**

Cells were fixed using 4% paraformaldehyde for 15 minutes. After fixation, cells were treated with 0.1% Triton X-100 for 15 minutes at room temperature (RT). To prevent non-specific binding, cells were blocked with 5% BSA for 1 hour at RT. Then cells were incubated with 100 µl of anti-HNRNPC antibody at 4°C overnight. The following day, cells were washed three times with phosphate-buffered saline (PBS) to remove unbound primary antibodies. The cells were then incubated with the corresponding secondary conjugated-antibody, for 1 hour at RT. DAPI was used to stain the cell nucleus. Images were acquired using a fluorescence microscope from Olympus (Tokyo, Japan).

Immunohistochemistry was performed on human renal carcinoma tissue samples to detect and visualize specific proteins in the tissue. 4 µm thick slides of ccRCC tissues were dewaxed and dehydrated to prepare the tissue sections for antibody staining. The tissue sections were treated with 3% hydrogen peroxide (H_2_O_2_) for 10 minutes at RT to block endogenous peroxidase activity, which can interfere with the staining process. Then the tissue slides were blocked with 5% goat serum for 30 minutes at RT to prevent non-specific binding of antibodies and incubated with a rabbit/mouse polyclonal antibody specific to the target protein at 4°C overnight. The next day, the slides were washed with PBS three times to remove unbound primary antibodies. Then tissue sections were incubated with a biotinylated rabbit/mouse anti-rabbit/mouse IgG secondary antibody for 1 hour at 37°C. After washing, the tissue samples were stained with 3,3'-diaminobenzidine (DAB) substrate, resulting in a brown-colored precipitate at the sites where the primary antibody bound. The images of the stained tissue slides were obtained using a microscope from Olympus (Japan).

**In vitro cell assays**

For cell proliferation assay, 10^3^ cells were initially seeded into individual wells of 96-well plates and allowed to attach for 24 hours. The cell proliferation assay was conducted over 5 consecutive days. The Cell Counting Kit-8 assay (APExBIO, USA) was employed to assess cell viability based on the manufacturer's instructions. For Edu assays, the EdU Flow Cytometry Assay Kits (Cy3) (APExBIO, USA) was utilized following the kit instructions. For the colony formation assay, 10^3^ cells were seeded in 2 ml complete culture media in six-well plates. Cells were allowed to grow for two weeks. After the incubation period, colonies were stained using Crystal Violet, and their numbers were quantified. For migration and invasion assays, Transwell filter chambers (8 μm pore size; Corning, NY) with or without Matrigel (Corning, NY) were used according to the manufacturer's instructions. Five random fields from each well were counted under a microscope to evaluate the migration and invasion potential of the cells. For the wound healing assay, cells were seeded in 6-well plates and grown until approximately 80% confluence. A sterile pipette tip was used to create a scratch line on the cell monolayer. The photos were taken after 24 hours, and the migration rate was calculated using ImageJ software.

**In vivo tumorigenesis and metastasis assays**

For tumorigenesis assay, 6-week-old female BALB/c nude mice were selected for the xenograft experiments. The Caki-1 cells were transfected with either pLenti-Vector or pLenti-shcircPPAP2B plasmid to modify their gene expression. After being selected with Puromycin (2μg/ml) for 2 weeks, 5×10^6^ Caki-1 cells were subcutaneously injected into the nude mice. Once a palpable tumor was formed, its size was measured weekly using a caliper. The tumor volume was calculated using the formula: volume = length × width^2^ × 0.5. For metastasis assay, 2×10^6^ Caki-1cells were injected into the tail vein of nude mice. Eight weeks after injection for circPPAP2B knockdown metastasis assay and six weeks after injection for circPPAP2B overexpression metastasis assay, the mice were sacrificed. Lung tissues were then collected and fixed using a 4% phosphate-buffered formalin solution. The lung tissues were analyzed by HE staining to identify and visualize metastatic tumor burden. The study was approved by the Ethics Committee of Nanfang Hospital, Southern Medical University (IACUC-LAC-20220425-003).

**RNA pulldown assays and Mass spectrometry analysis**

For the RNA pull-down assay, a biotin-labeled sense probe was specifically designed to bind to the junction site of circPPAP2B. An antisense probe was used as a control in this experiment. Approximately 10^7^ cells were washed with ice-cold PBS and lysed in lysis buffer or EBC buffer supplemented with a cocktail of proteinase inhibitors, phosphatase inhibitors, and RNase inhibitors from Invitrogen. The cell lysates were incubated with 3μg of biotinylated probes at room temperature for 2 hours. Streptavidin magnetic beads from Life Technologies (USA) were used to pulldown the target biotin-coupled RNA complex. Before the pulldown assay, the beads were washed in lysis buffer three times. The washed beads were added to the cell lysates and further incubated for another 4 hours at room temperature. Subsequently, the beads were separated, and the bound proteins were analyzed using mass spectrometry analysis. Additionally, western blot analysis was performed to validate the presence of specific proteins of interest. Probe sequences are listed in **Supplementary Table 7**.

**RNA immunoprecipitation (RIP) assays**

Approximately 2 × 10^7^ ccRCC cells were lysed in RIPA lysis buffer or EBC containing a cocktail of proteinase inhibitors and phosphatase inhibitors. A small portion (1/100) of the cell lysates was set aside as the input control and stored at -80 °C for subsequent total RNA extraction. The remaining cell lysates were divided into two groups. One group was incubated with 4μg of anti-HNRNPC antibody to immunoprecipitate the HNRNPC-RNA complexes, while the other group was incubated with IgG as a negative control. After overnight incubation at 4 °C, the lysates were washed with NETN buffer three times to remove non-specifically bound proteins. Protein A/G sepharose beads were then added to each group and incubated with the antibody-protein complexes at 4 °C for 4 hours. Following the incubation with the beads, the RIP complexes and the input control were separately incubated with proteinase K buffer for 45 minutes at 50 °C. The RNA from the RIP complexes and the input control was then extracted using Glycogen precipitation. The extracted RNA was subjected to qPCR analysis to investigate the presence and enrichment of specific RNA sequences bound to the HNRNPC.

**MeRIP assays**

Total RNAs were extracted from the cells, and one-tenth of the total RNAs were saved as the input control. Protein A/G sepharose beads were separately conjugated with 5 μg of IgG (a negative control) and anti-m6A antibody (S68055-1-Ig, Proteintech). The prepared Protein A/G sepharose beads were then incubated with the total RNAs in an immunoprecipitation buffer containing an RNase inhibitor. The incubation was carried out at 4 °C overnight. During this step, the anti-m6A antibody selectively binds to the m6A-modified RNA molecules present in the total RNA pool. After the overnight incubation, the methylated RNA bound to the anti-m6A antibody was eluted by competition with free m6A, ensuring the specific recovery of m6A-modified RNA from the immunoprecipitation reaction. The eluted m6A-modified RNA was recovered and subjected to qPCR analysis, along with the input control. The enrichment of m6A in each sample was calculated by normalizing the qPCR results to the corresponding input control.

**Co-immunoprecipitation (CoIP) assays**

Approximately 10^7^ cells were harvested and lysed using RIPA buffer supplemented with protease inhibitors. Five percent of the cell lysate was set aside as the input control. The remaining cell lysate was then incubated with primary antibody overnight at 4°C. Then the cell lysate was incubated with protein A/G beads for 6 hours at 4°C. After the incubation, the bead-protein complexes were washed three with NETN buffer to remove non-specifically bound proteins, ensuring specificity in the subsequent steps. Then the proteins were removed from the beads with a loading buffer under standard denaturing conditions. Purified proteins were detected by Western blotting.

**Dual-luciferase reporter assay**

Two versions of the circPPAP2B sequence were created: the wild-type (WT) and the mutant (Mut) sequences. These sequences were inserted into the dual luciferase reporter vector pSICHECK2. ccRCC cells were co-transfected with the wild-type or mutant luciferase reporter plasmids and either miRNA mimics or a negative control with Lipofectamine 3000 (Thermo). After 48 hours of transfection, the cells were allowed sufficient time for the regulatory interactions between miRNAs and circPPAP2B to take place. The firefly and renilla luciferase activities in the transfected cells were measured using the Luciferase Reporter Gene Assay Kit from Yeasen Biotechnology (Product No. 11401ES60). The values of luminescence from the firefly and renilla luciferases were obtained separately. Then, the ratio of luminescence from firefly to renilla luciferase was calculated for each group.

**Statistics**

All data were shown as mean ± SEM. The Student t-test was used to compare the difference in gene expression between the two groups. ANOVA was used to compare the difference in gene expression among multiple groups. Pearson correlation coefficient assay was used to analyze the expression correlation. Log-rank test was used to assess survival differences. All statistical tests were two-sided.
